# Unraveling the Molecular Mechanisms Underlying Complement Dysregulation by Nephritic Factors in C3G and IC-MPGN

**DOI:** 10.3389/fimmu.2018.02329

**Published:** 2018-10-15

**Authors:** Roberta Donadelli, Patrizia Pulieri, Rossella Piras, Paraskevas Iatropoulos, Elisabetta Valoti, Ariela Benigni, Giuseppe Remuzzi, Marina Noris

**Affiliations:** ^1^Clinical Research Center for Rare Diseases Aldo e Cele Daccò and Centro Anna Maria Astori, Science and Technology Park Kilometro Rosso, Istituto di Ricerche Farmacologiche Mario Negri IRCCS, Bergamo, Italy; ^2^Unit of Nephrology and Dialysis, Azienda Socio-Sanitaria Territoriale Papa Giovanni XXIII, Bergamo, Italy; ^3^Department of Biomedical and Clinical Sciences, University of Milan, Milan, Italy

**Keywords:** C3 glomerulopathy, membranoproliferative glomerulonephritis, complement alternative pathway, C3 nephritic factors, terminal complement complex, C3 convertase, factor H

## Abstract

Membranoproliferative glomerulonephritis (MPGN) was recently classified as C3 glomerulopathies (C3G), and immune-complex (IC) mediated MPGN. Dysregulation of the complement alternative pathway, driven by acquired and/or genetic defects, plays a pathogenetic role in C3G. However, alternative pathway abnormalities were also found in IC-MPGN. The most common acquired drivers are the C3 nephritic factors (C3NeFs), heterogeneous autoantibodies that stabilize the C3 convertase, C3bBb. C3NeFs are traditionally detected by hemolytic assays based on sheep erythrocyte lysis, which however do not provide a direct molecular estimation of C3bBb formation and decay. We set up a microplate/western blot assay that specifically detects and quantifies C3bBb, and its precursor, the C3 proconvertase C3bB, to investigate the complex mechanistic effects of C3NeFs from patients with primary IC-MPGN (*n* = 13) and C3G (*n* = 13). In the absence of properdin, 9/26 patients had C3NeF IgGs stabilizing C3bBb against spontaneous and FH-accelerated decay. In the presence of properdin the IgGs of all but one patient had C3bBb-stabilizing activity. Properdin-independent C3NeFs were identified mostly in DDD patients, while properdin-dependent C3NeFs associated with either C3GN or IC-MPGN and with higher incidence of nephrotic syndrome. When we grouped patients based on our recent cluster analysis, patients in cluster 3, with highly electron-dense intramembranous deposits, low C3, and mostly normal sC5b-9 levels, had a higher prevalence of properdin-independent C3NeFs than patients in clusters 1 and 2. Conversely, about 70% of cluster 1 and 2 patients, with subendothelial, subepithelial, and mesangial deposits, low C3 levels and high sC5b-9 levels, had properdin-dependent C3NeFs. The flexibility of the assay allowed us to get deep insights into C3NeF mechanisms of action, showing that: (1) most C3NeFs bind strongly and irreversibly to C3 convertase; (2) C3NeFs and FH recognize different epitopes in C3 convertase; (3) C3NeFs bind rapidly to C3 convertase and antagonize the decay accelerating activity of FH on newly formed complexes; (4) C3NeFs do not affect formation and stability of the C3 proconvertase. Thus, our study provides a molecular approach to detecting and characterizing C3NeFs. The results highlight different mechanisms of complement dysregulation resulting in different complement profiles and patterns of glomerular injury, and this may have therapeutic implications.

## Introduction

Membranoproliferative glomerulonephritis (MPGN) is a rare chronic kidney disorder associated with thickening of the glomerular capillary wall and mesangial expansion, which are the result of the deposition of immune-complexes and complement factors (1, 2). Evidence of the participation of the third fraction of complement C3 in this type of lesion, dates back to the middle of the last century (3, 4). Traditionally, on the basis of electron microscopy localization of electron-dense deposits relative to the glomerular basement membrane, MPGN was divided into type I, with subendothelial deposits (5), type II or Dense-Deposit Disease (DDD), with intramembranous highly electron-dense deposits (6), and type III, with subendothelial and subepithelial deposits (7).

An advanced approach toward an etiology-based diagnosis of this disease arose from the more recent immunofluorescence (IF)-based classification that distinguishes between immune-complex-mediated MPGN (IC-MPGN), with glomerular IgG and C3 deposits, and C3 glomerulopathy (C3G) with predominant C3 deposits (8, 9). C3G is further divided into DDD, with distinctive highly electron-dense deposits within the glomerular basement membrane; and C3 glomerulonephritis (C3GN), with mesangial, intramembranous, subendothelial and sometimes subepithelial deposits (1, 6, 10, 11). According to the current classification, IC-MPGN derives from the deposition of immune-complexes that form in the context of infections, autoimmune diseases and malignancies, and trigger the classical complement pathway (8). C3G instead arises from abnormalities in the control of the complement alternative pathway (8). However the distinction of IC-MPGN as an immune-complex mediated disease and C3G as an alternative pathway-mediated disease leaves a number of questions open. In up to 25–30% of cases classified as IC-MPGN based on the IF finding, an underlying condition could not be identified (11, 12). In addition, in about 16% of patients undergoing repeated kidney biopsies the diagnosis shifted from IC-MPGN to C3G and vice versa (11, 13). Finally, genetic and acquired alternative pathway abnormalities have been found as frequently in patients with IC-MPGN as in those with C3G (14–16), suggesting that dysregulation of the complement alternative pathway may play a role in both conditions.

The most common acquired drivers in primary IC-MPGN and C3G are the C3 nephritic factors (C3NeFs) (17–20) a heterogeneous group of autoantibodies that bind to neoepitopes of the C3 convertase complex C3bBb, the key amplifying enzyme of the complement alternative pathway that cleaves C3 to C3a and C3b (21, 22). C3NeFs stabilize both the fluid-phase and the cell-bound C3 convertase (23), markedly increasing its half-life and inhibiting the accelerated decay mediated by complement regulators such as factor H (FH), decay-accelerating factor (DAF) and complement receptor 1 (CR1) (20, 24, 25). C3NeFs are present in 40-50% of patients with IC-MPGN or C3GN and in 70–80% of patients with DDD (14, 16, 26).

A consensus regarding the detection and characterization of C3NeFs is lacking. Few specialized laboratories routinely measure C3NeFs using different methods: they predominantly use hemolytic assays based on the evaluation of the lysis of sheep erythrocytes carrying preformed alternative pathway C3 convertase (27). There is still uncertainty regarding the pathogenetic role of C3NeFs in IC-MPGN and C3G. C3NeF activity, as measured by the hemolytic assays correlates poorly with disease activity and with circulating complement parameters. Some C3NeFs induce C3 consumption in the fluid phase, but do not always enhance the activation of the terminal pathway (28, 29), other C3NeFs are even associated with normal or near normal C3 levels (30). Recent studies have explored the mechanisms of action of C3NeFs in patients with C3G using several functional assays based on ELISA (31), surface plasmon resonance (31) and modified hemolytic assays (26, 28), which confirmed the heterogeneous nature of C3NeFs. Some C3NeFs were found to require the presence of properdin to carry out their stabilizing activity on the C3 convertase (26, 28, 31) and were called C5NeFs since they also activated the terminal complement pathway, while other C3NeFs were properdin-independent and had no effect on C5-cleavage. In one study, some correlation was found with complement profile and histology diagnosis: indeed, properdin-dependent C3NeFs (C5NeFs) were more frequent in patients with C3GN and high plasma levels of the soluble terminal complement complex sC5b-9, while properdin-independent C3NeFs were associated with DDD and complement activation restricted to the C3 level (28). However, electron microscopy examination was lacking in about half of patients, which questions regarding the distinction between C3GN and DDD. In addition, the above published studies did not include patients with IC-MPGN.

In this study we set up a user-friendly method that specifically detects and quantifies the alternative pathway C3 convertase, C3bBb, and its precursor, the C3 proconvertase C3bB, to investigate the complex mechanistic effects of C3NeFs in the pathogenesis of complement dysregulation in patients with IC-MPGN and C3G. The specific aims were: (1) to characterize and quantify C3NeF activity in stabilizing C3 convertase and its FH-mediated accelerated decay; (2) to investigate the effect of properdin on the activity of C3NeFs; (3) to evaluate whether the results of C3NeFs assays correlate with complement profile, clinical features and histological diagnosis.

Through unsupervised hierarchical clustering we recently identified, among IC-MPGN and C3G patients, four clusters, indicating the existence of different pathogenetic patterns underlying complement activation and glomerular injury (15). A large majority of patients carrying C3NeFs, as measured by the hemolytic assay, were included in clusters 1–3, while patients in cluster 4 had a very low prevalence of C3NeFs. In addition, clusters 1–2 included patients with alternative pathway activation at the C3 and C5 levels, as highlighted by low serum C3 and high plasma sC5b9 levels; while patients in cluster 3 had alternative pathway activation mainly at the C3 level (15). Thus, a further aim of this study was to use the new method to compare the activity of C3NeFs between patients in clusters 1–3.

Finally we investigated the mechanisms by which C3NeFs antagonize the FH-mediated regulatory activity on C3 convertase and whether C3NeFs had any effect on the formation and decay of the C3 proconvertase, issues that have not been investigated in depth so far.

## Materials and methods

### Patients and controls

Twenty-six patients with primary IC-MPGN or C3G were selected from the previously described cohort (15) based on positivity for C3NeF by hemolytic assay (>20%) (27), as well as on the availability of suitable amounts of serum samples collected outside of treatment with steroids, immunosuppressive drugs or eculizumab.

A diagnosis of MPGN was established in patients with the typical light microscopy pattern with mesangial hypercellularity, endocapillary proliferation, and capillary wall remodeling (1). Patients with glomerular immunoglobulin and C3 deposits at IF were considered IC-MPGN (8). C3G was diagnosed based on the presence of MPGN or mesangial proliferative patterns with “dominant C3” glomerular staining (C3c staining intensity at least two orders of magnitude greater than any other immune-reactant including IgG, IgM, IgA, and C1q on a 0 to 3 scale) (10). Based on electron microscopy, C3G was further classified as DDD or C3GN. Patients with MPGN secondary to autoimmune diseases, monoclonal gammopathy, infections (HBV, HCV and HIV), neoplasia or atypical hemolytic uremic syndrome were excluded.

Control sera were obtained from 26 donors with no history of renal disease. Twelve patients with primary IC-MPGN or C3G (IC-MPGN, *n* = 5; C3GN, *n* = 4; DDD, *n* = 3) without C3NeF activity by hemolytic assay were also studied as additional controls. The above patients were selected to be homogenously distributed among clusters 1 to 4 (*n* = 3 for each cluster) (15).

All participants provided informed written consent. The study adheres to the Declaration of Helsinki and was approved by the Ethics Committee of the Azienda Sanitaria Locale of Bergamo (Italy).

### Serum and plasma complement profile and genetic analysis

Complement C3 levels in serum were measured by nephelometry. SC5b-9 and C3d levels were evaluated in plasma by MicroVue sC5b-9 Plus EIA (sC5b-9 Plus; Quidel), and ELISA (Human complement C3d ELISA kit Assaypro LLC), respectively. For the latter tests, blood was collected in ice-cold EDTA tubes and immediately centrifuged at 4°C to avoid *ex vivo* complement activation. Plasma was quickly separated and frozen at −80°C until assayed. Normal ranges were defined as follows: C3, 90–180 mg/dl (mean ± 2 SD of the laboratories of the ASST Papa Giovanni XXIII, Bergamo, Italy); sC5b-9, 127–400 ng/ml (mean ± 2 SD of 50 healthy control subjects); C3d/C3 ratio, 0.01-0.09 (mean ± 2SD of 15 healthy control subjects).

Screening for *CFH, MCP, CFI, CFB, C3* and *THBD* coding sequences was performed by amplicon-based next generation sequencing (14). Rare functional variants (missense, nonsense, indel, or splicing variants with minor allele frequency, MAF <0.001 in 1,000 Genomes and ExAC databases) were selected and defined as likely pathogenetic, or pathogenetic when published functional studies were available. Anti-FH autoantibodies were measured in plasma by ELISA (32).

### Selective C3bB C3 proconvertase and C3bBb C3 convertase formation and decay assays

For the generation of alternative pathway C3bB C3 proconvertase and C3bBb C3 convertase we first tried an ELISA assay previously described by Hourcade et al. (33, 34). ELISA plates (Nunc-Immuno Maxisorp) coated with 3 μg/ml C3b (Complement Technology Inc., Tyler, TX) were incubated at 37°C for 2 h with different concentrations of FB (0–500 ng/ml, Complement Technology Inc.) in the absence or in the presence of FD (25 ng/ml, Complement Technology Inc.), respectively, both diluted in assay buffer (8.1 mM Na_2_HPO_4_, 1.8 mM NaH_2_PO_4_, 4% BSA, 0.1% Tween20, and 75 mM NaCl) containing 2 mM NiCl_2_. After washes with wash buffer (8.1 mM Na_2_HPO_4_, 1.8 mM NaH_2_PO_4_, 0.1% Tween20 and 25 mM NaCl) supplemented with 2 mM NiCl_2_, the C3bB and C3bBb complexes were detected by ELISA using polyclonal goat anti-human FB antibody (Quidel, San Diego, CA) diluted 1:10,000, followed by HRP-conjugated anti-goat antibody (1:40,000; Sigma Aldrich), both diluted in antibody buffer (8.1 mM Na_2_HPO_4_, 1.8 mM NaH_2_PO_4_, 4% BSA, 0.1% Tween20 and 25 mM NaCl) supplemented with 2 mM NiCl_2_. Color was developed using 3,3′, 5, 5′-teramethylbenzidine (TMB) substrate (Tema Ricerca srl, Bologna, Italy) and stopped with H_2_SO_4_ 2 M, and absorbance was measured at 450 nm. Each reaction was performed in duplicate and the OD values averaged. As shown in Supplementary Figure 1A, the ELISA curves from the reaction of coated C3b with either FB alone or FB plus FD showed dose-dependent superimposable profiles, indicating that the ELISA assay cannot selectively discriminate between C3bB and C3bBb formation. To specifically generate either C3bB or C3bBb complexes, we exploited the selective stabilization abilities of Mn^2+^and Mg^2+^, respectively (35, 36). For the generation of C3bB(Mn^2+^) or C3bBb(Mg^2+^) complexes, C3b-coated microtiter wells were treated as above, except that the incubation was performed in the presence of 2 mM MnCl_2_ for 2 h at 37°C (Supplementary Figure 1B) or 10 mM MgCl_2_ for 30 min at 25°C (Supplementary Figure 1C), respectively. In either condition the amount of the complexes formed was too small to be detected by ELISA, likely due to dissociation of FB and Bb from C3bB(Mn^2+^) and the C3bBb(Mg^2+^) respectively, during post-reaction incubations with primary and secondary antibodies and the intercurrent washing steps.

Thus we used a combined microplate and western blot (WB) technique to specifically detect C3bB and C3bBb (37). Briefly, C3bB(Mn^2+^) complexes were formed by incubating C3b-coated wells at 37°C for 2 h with FB (1,000 ng/ml), diluted in the assay buffer (8.1 mM Na_2_HPO_4_, 1.8 mM NaH_2_PO_4_, 0.1% Tween 20, and 75 mM NaCl) supplemented with 4% BSA, and 2 mM MnCl_2_. C3bBb(Mg^2+^) complexes were formed by incubating C3b-coated wells at 25°C for 12 min with FB (1,000 ng/ml) and FD (10 ng/ml,) both diluted in assay buffer supplemented with 0.5% BSA and 10 mM MgCl_2_. After washes, the protein complexes were detached from microtiter wells with EDTA 10 mM and SDS 1% for 1 h, subjected to 10% SDS-PAGE, and transferred by electroblotting to Hybond-P hydrophobic polyvinylidene difluoride (PVDF) membrane (Amersham Biosciences, GE Healthcare, Euroclone Spa, Milano Italy). Proteins were detected with a polyclonal goat anti-human FB antibody (Quidel, San Diego, CA) followed by HRP-conjugated anti-goat Ab (Sigma Aldrich) and the ECL system (Amersham Biosciences, GE Healthcare, Euroclone Spa, Milano Italy). C3-proconvertase and C3-convertase formation were evaluated by the visualization by WB of the FB (93 kDa) or the Bb band (60 kDa), respectively (Supplementary Figure 1D). Images were acquired by the Odyssey Imager instrument (Licor).

For the generation of C3bB(Ni^2+^) and C3bBb(Ni^2+^) complexes, C3b-coated wells were incubated at 37°C with FB (1,000 ng/ml) and FD (10 ng/ml) in assay buffer containing 2 mM NiCl_2_ for 30 min. Then the Ni^2+^-protein complexes formed in the presence or in the absence of FD were evaluated by WB as described above. In the presence of FD, both FB (93 KDa) and Bb (60 KDa) bands were visualized (Supplementary Figure 1D) indicating that only a portion of C3bB was converted to C3bBb.

The intensity of the bands detected in WB was estimated by densitometry using NIH Software ImageJ (NIH, USA).

To study spontaneous or FH-mediated decay of C3 convertase and C3 proconvertase over time, C3bBb(Mg^2+^) and C3bB(Mn^2+^) were allowed to form for 12 min at 25°C and 2 h at 37°C, respectively, as reported above. In some wells the formed complexes were detached from microtiter wells with EDTA 10 mM and SDS 1%, subjected to WB analyses as described above and used as baseline.

Additional wells were then washed and the formed complexes were incubated with selective assay buffers in the presence or in the absence of FH (2,640 ng/ml; physiological molar ratio FB:FH = 1:1.6; Merck) for the following time periods: C3bBb(Mg^2+^), 2, 8, 16, and 32 min; C3bB(Mn^2+^), 30, 60, 120, and 240 min.

To study the spontaneous or FH-mediated decay of C3bB and C3bBb simultaneously, C3bB(Ni^2+^) and C3bBb(Ni^2+^) were formed together during a 30 min period at 37°C. In some wells the formed complexes were detached from microtiter wells with EDTA 10 mM and SDS 1%, subjected to WB analyses, and used as baseline. Additional wells were then washed and spontaneous and FH-mediated decay of the formed complexes were monitored by incubating the wells at 37°C with assay buffer alone or with FH (2,640 ng/ml) for 5, 30, 60, and 120 min. Following washes, the remaining complexes were detached from microtiter wells with EDTA 10 mM and SDS 1% and subjected to WB analyses. The percentage of residual Bb or B band was calculated as the ratio of the densities (in Pixel^2^) of the Bb or B bands after decay and the corresponding baseline Bb or B band density before decay x 100.

### C3NeF C3 convertase-stabilizing activity assays

IgGs were isolated from sera by the Melon Gel IgG Purification Kit (Thermo Scientific, VWR International PBI srl, Milano Italy)

To evaluate the ability of C3NeF IgG to stabilize the alternative pathway C3 convertase, C3bBb, against spontaneous and FH-mediated decay, we adapted the microplate/WB described above. Three protocols were set up. Each C3NeF IgG sample was analyzed three times.

**Protocol 1**. C3b-coated wells were incubated for 12 min at 25°C with 1,000 ng/ml FB, 10 ng/ml FD, 100 μg/ml IgGs purified from patients or controls, and 10 mM MgCl_2_, in the absence or in the presence of 2,640 ng/ml FH. In some wells the formed complexes were detached with EDTA 10 mM and SDS 1%, and subjected to WB analyses. The amount of C3bBb formed in the absence of FH was used as the baseline.

In additional wells, the complexes formed in the absence of FH were washed and C3NeF IgG C3 convertase-stabilizing activity (CSA) was evaluated by incubation for 32 min at 25°C with buffer alone (sCSA) or with buffer containing 2,640 ng/ml FH, (FH-CSA). Following washes, the residual C3bBb C3 convertase complexes were detached from microtiter wells with EDTA 10 mM and SDS 1% and subjected to WB analyses. The intensity of the band detected in WB was estimated by densitometry using NIH Software ImageJ (NIH, USA). The percentage of residual Bb band was calculated as the ratio of the densities (in Pixel^2^) of each Bb band after decay and the corresponding baseline Bb band density before decay x 100.

**Protocol 2**. C3b coated wells were incubated for 12 min at 25°C with 1,000 ng/ml FB, 10 ng/ml FD, and 10 mM MgCl_2_. After washing, the complexes formed were incubated for 32 min at 25°C in the presence of 100 μg/ml IgGs purified from patients or healthy controls without (spontaneous decay) or with (FH-mediated decay) 2,640 ng/ml FH. Following washes, the residual C3bBb C3 convertase complexes were detached from microtiter wells. The C3NeF IgGs C3 convertase stabilizing activity was calculated as the ratio of the densities (in Pixel^2^) of Bb bands after decay in the presence of patient IgGs and the Bb bands after decay in the presence of control IgGs.

**Protocol 3**. The C3 convertase-stabilizing activity assay with properdin (PCSA) assay, is similar to the CSA, with the only difference that properdin (P; purified from human serum, Complement Technology Inc.) is included in the protocol during C3 convertase formation. Briefly, C3b-coated wells were incubated for 12 min at 25°C with 1,000 ng/ml FB, 10 ng/ml FD, 100 μg/ml IgGs purified from patients or healthy controls and 10 mM MgCl_2_, in the presence of 500 ng/ml properdin (Complement Technology Inc.). The C3 convertase formed, C3bBbP, was allowed to decay in the absence (sPCSA) or in the presence of FH (FH-PCSA) for 32 min at 25°C. After washes, the following steps were identical to those described above. The percentage of residual Bb band was calculated as the ratio of the densities (in Pixel^2^) of each Bb band after decay and the corresponding baseline Bb band density before decay x 100. For FH detection, WB membranes from all three protocols were labeled with a goat anti-FH antibody (Calbiochem, Billerica, MA, dil 1:10,000) followed by HRP-conjugated anti-goat Ab (Sigma Aldrich, dil 1:10,000) and the ECL system (Amersham Biosciences, GE Healthcare).

### Effect of C3NeF on C3bB(Mn^2+^), C3bB(Ni^2+^), and C3bbb(Ni^2+^) decay

C3b-coated wells were incubated for 2 h at 37°C with 1,000 ng/ml FB, and 2 mM MnCl_2_ in the presence of 100 μg/ml IgGs purified from patients P5 and P10 or healthy controls. In some wells the formed complexes were detached from microtiter wells with EDTA 10 mM and SDS 1%, and subjected to WB analyses as described above. In additional wells, the C3bB proconvertase formed, C3bB(Mn^2+^), was allowed to decay in the absence or in the presence of FH for 30, 60, 120, and 240 min at 37°C. Following washes, the residual C3bB(Mn^2+^) complexes were detached and subjected to WB analyses. The intensity of the B band detected in WB was estimated by densitometry using NIH Software ImageJ (NIH, USA). The percentage of residual B band was calculated as the ratio of the densities (in Pixel^2^) of each B bands after decay and the corresponding baseline B band density before decay x 100.

C3bB(Ni^2+^) and C3bBb(Ni^2+^) were formed together during a 30 min period at 37°C, incubating C3b-coated wells with 1,000 ng/ml FB, 10 ng/ml FD, and 2 mM NiCl_2_ without (baseline) or with 2,640 ng/ml FH in the absence or in the presence of 100 μg/ml IgGs purified from patients P5 and P10 or healthy controls. In some wells the formed complexes were detached from microtiter wells with EDTA 10 mM and SDS 1%, subjected to WB analyses, and used as baseline. Additional wells were washed and spontaneous and FH-mediated decay of the complexes formed without FH were monitored by incubating the wells at 37°C with assay buffer alone or with FH (2,640 ng/ml) for 30 min. Following washes, the remaining complexes were detached from microtiter wells and subjected to WB analyses. The percentage of residual Bb or B band was calculated as the ratio of the densities (in Pixel^2^) of the Bb or B bands after decay and the corresponding baseline Bb or B band density before decay x 100.

For FH detection, WB membranes were labeled with a goat anti-FH antibody followed by HRP-conjugated anti-goat Ab and the ECL system.

### Statistical analysis

Continuous variables were analyzed by ANOVA. The Fisher Exact test was used for categorical variables. Linear regression analysis was used for correlations of continuous variables. *P* values < 0.05 were considered to be statistically significant. Analyses were performed using the R platform v.3.5.0, and the MedCalc v.12.2.1.0 software. The intra and inter-assay coefficients of variation were calculated as SD/mean x 100.

## Results

### Description of patients

The clinical, histologic and biochemical data of the 26 C3NeF^+^ patients are shown in Table 1. According to the IF-based classification, 13 patients had IC-MPGN and 13 had C3G (C3GN *n* = 4, DDD, *n* = 9). Age of onset ranged from 4.9 to 30.6 years and did not differ among histology groups (C3GN: 12.6±4.2; DDD: 12.2 ± 5.4; IC-MPGN: 12.9 ± 6.8 years). Males accounted for 50, 56, and 54% of the C3GN, DDD, and IC-MPGN patients, respectively. At onset all patients had hematuria (micro or gross hematuria) and/or proteinuria. Urinary protein excretion levels did not significantly differ between histology groups (C3GN: 1.8 ± 1.4; DDD: 2.4 ± 2.6; IC-MPGN: 4.7 ± 4.4.g/24 h). Renal function at onset was normal or near normal in all patients (serum creatinine: C3GN: 0.68 ± 0.2; DDD: 0.56 ± 0.2; IC-MPGN: 0.72 ± 0.2 mg/dl) (Table 1). C3 levels at onset were low and did not differ between histology groups (C3GN: 40 ± 11; DDD: 19 ± 19; IC-MPGN: 21 ± 15 mg/dl), while plasma sC5b-9 levels were higher in C3GN (1249 ± 736 ng/ml) and IC-MPGN (1895 ± 1291 ng/ml) than in DDD (416 ± 374 ng/ml, *P* < 0.01) (Table 1). No significant correlation was found between serum C3 (r = −0.31, *P* = 0.15) or plasma sC5b-9 (r = 0.30, *P* = 0.15) levels and urinary protein excretion at onset. Complement gene likely pathogenetic variants were identified in 4 patients (1 in *CFH*, 2 in ***CFB*, **and 1 in *THBD*, Table 2). Anti-FH autoantibodies were identified in two patients (P26 with homozygous *CFHR3/CFHR1* deletion and P15 with two copies of *CFHR3/CFHR1*) (Table 2). All patients were positive for C3NeF by the hemolytic assay, as per inclusion criteria (Table 2).

**Table 1:** Clinical, biochemical and histologic parameters of patients.

			**Clinical and biochemical data at onset**						**Glomerular IF**			**Glomerular deposits on EM**			
**Patients**	**Histology**	**Cluster**	**Age (y)**	**Hematuria**	**UProt g/24 h**	**sCreat (mg/dl)**	**Serum C3 (mg/dl)**	**Plasma SC5b-9 (ng/ml)**	**C3**	**IgG**	**C1q**	**Mesangial**	**Subepi-thelial**	**Subendo-thelial**	**Intramembr highly dense**
P6	C3GN	1	8	GrossH	1.0	0.4	40	1298	3+	1+	Neg	No	Yes	Yes	No
P17	C3GN	1	15	MicroH	3.8	0.8	24	2270	2+/3+	Neg	Neg	Yes	No	Yes	No
P21	C3GN	1	17	MicroH	1.3	0.8	50	656	3+	Neg	Neg	Yes	Yes	Yes	No
P24	C3GN	1	10.2	MicroH	1.0	0.7	44	772	3+	Neg	Neg	Yes	Yes	Yes	No
P3	IC-MPGN	1	14.9	MicroH	0.3	0.8	11	4488	3+	3+	Neg	Yes	Yes	Yes	No
P12	IC-MPGN	1	11.4	MicroH	3.9	0.4	17	1391	3+	Neg*	1+	No	Yes	Yes	No
P13	IC-MPGN	1	16.3	MicroH	9.5	0.7	13	2086	3+	Neg	2+	No	Yes	Yes	No
P30	IC-MPGN	1	18.6	No	2.5	0.9	40	1874	3+	2+	1+	Yes	Yes	Yes	No
P29	IC-MPGN	2	5.3	MicroH	0.5	0.6	36	1600	3+	3+	Neg	No	Yes	Yes	No
P8	IC-MPGN	2	30.6	MicroH	Pos**	1.0	45	249	3+	3+	2+	No	No	Yes	No
P11	IC-MPGN	2	9.2	MicroH	3.0	0.7	10	3520	3+	3+	1+	Yes	No	Yes	No
P14	IC-MPGN	2	12	MicroH	6.6	0.4	45	593	3+	2+	1+/2+	Yes	No	Yes	No
P16	IC-MPGN	2	11	MicroH	7.9	0.9	4	3365	3+	2+	1+	No	Yes	Yes	No
P18	IC-MPGN	2	8.5	GrossH	1.8	0.6	16	1643	3+	2+	2+	No	No	Yes	No
P28	IC-MPGN	2	7	MicroH	5.0	0.7	5	1080	2+/3+	2+	1+/2+	Yes	Yes	Yes	No
P25	IC-MPGN	2	17	MicroH	15.0	0.8	17	2474	3+	3+	3+	No	No	Yes	No
P15	IC-MPGN	3	6.5	GrossH	0.8	0.9	18	277	2+	1+	Neg	Yes	No	No	Yes
P5	DDD	3	12.3	MicroH	0.1	0.6	18	137	2+/3+	Neg	Neg	No	No	No	Yes
P9	DDD	3	24.7	No	0.4	0.6	54	286	2+/3+	Neg	Neg	Yes	No	No	Yes
P10	DDD	3	11.7	GrossH	4.4	0.9	5	380	3+	Neg	1+	Yes	No	No	Yes
P19	DDD	3	11.8	GrossH	5.0	0.5	9	267	3+	Trace	Neg	No	No	No	Yes
P20	DDD	3	4.9	GrossH	0.9	0.2	16	195	3+	Neg	Neg	Yes	No	No	Yes
P22	DDD	3	14	MicroH	Pos**	0.7	49	237	3+	1+	Neg	No	No	No	Yes
P23	DDD	3	8.1	GrossH	0.4	0.4	10	337	2+/3+	Neg	Trace	Yes	Yes	No	Yes
P26	DDD	3	10.6	GrossH	1.5	0.5	9	545	3+	Neg	1+	No	No	No	Yes
P2	DDD	1	11.3	GrossH	6.8	0.6	4	1364	3+	Neg	Trace	Yes	No	Yes	Yes

*GrossH, gross hematuria; MicroH, microhematuria. Uprot, urinary proteins; sCreat, serum creatinine*.

* IF IgM staining: 3+;

** Measured by dipstick.

*Normal values : Uprot, < 0.2 g/24.h; sCreat: < 1.3 mg/dl for adults, < 0.5 mg/dl in children < 5 years of age, and < 0.8 mg/dl in chidren >5 years; C3 levels.: 90–180 mg/dl; plasma sC5b-9 < 400 ng/ml*.

**Table 2:**
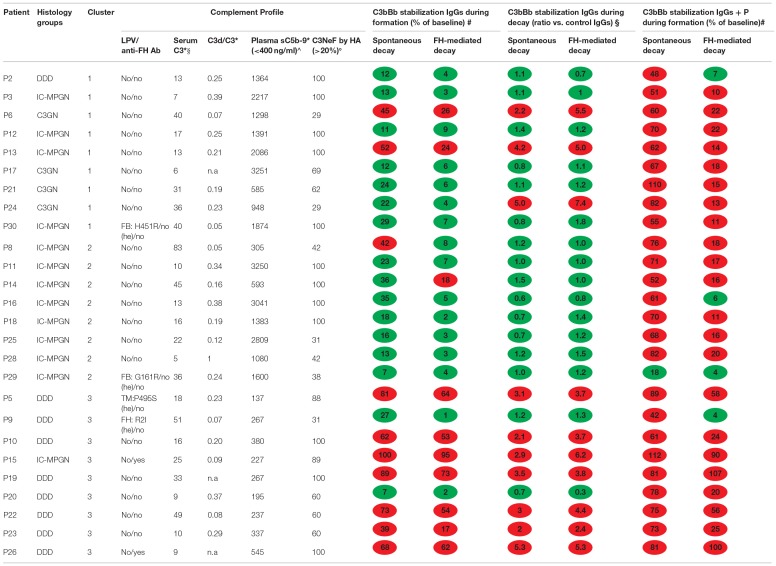
Complement profile and results of microplate/WB tests for C3NeF IgGs in the 26 patients with C3G or primary IC-MPGN.

LPV, likely pathogenetic variants; Ab, antibodies.

* Values measured at the time of sample collection of IgG isolation;

$ serum C3 normal range 90–180 mg/dl; °limit of C3NeF positivity in the hemolytic assay (HA);

∧ upper limit of normal plasma sC5b-9 range. WB, western blot; TM, thrombomodulin.

# IgGs were added during C3 convertase formation;

§ *IgGs were added during decay. Results are the mean of 3 experiments for each patient. n.a, sample not available*.

### Effect of C3NeFs from patients with C3G/IC-MPGN on AP C3 convertase decay

To evaluate the ability of C3NeF IgGs to stabilize the alternative pathway C3 convertase, C3bBb, and investigate the underlying molecular mechanisms, we developed a microplate/WB assay that specifically detects the Bb component of the C3bBb convertase, exploiting the selective stabilization properties of Mg^2+^ on C3bBb.

**Figure 1:**
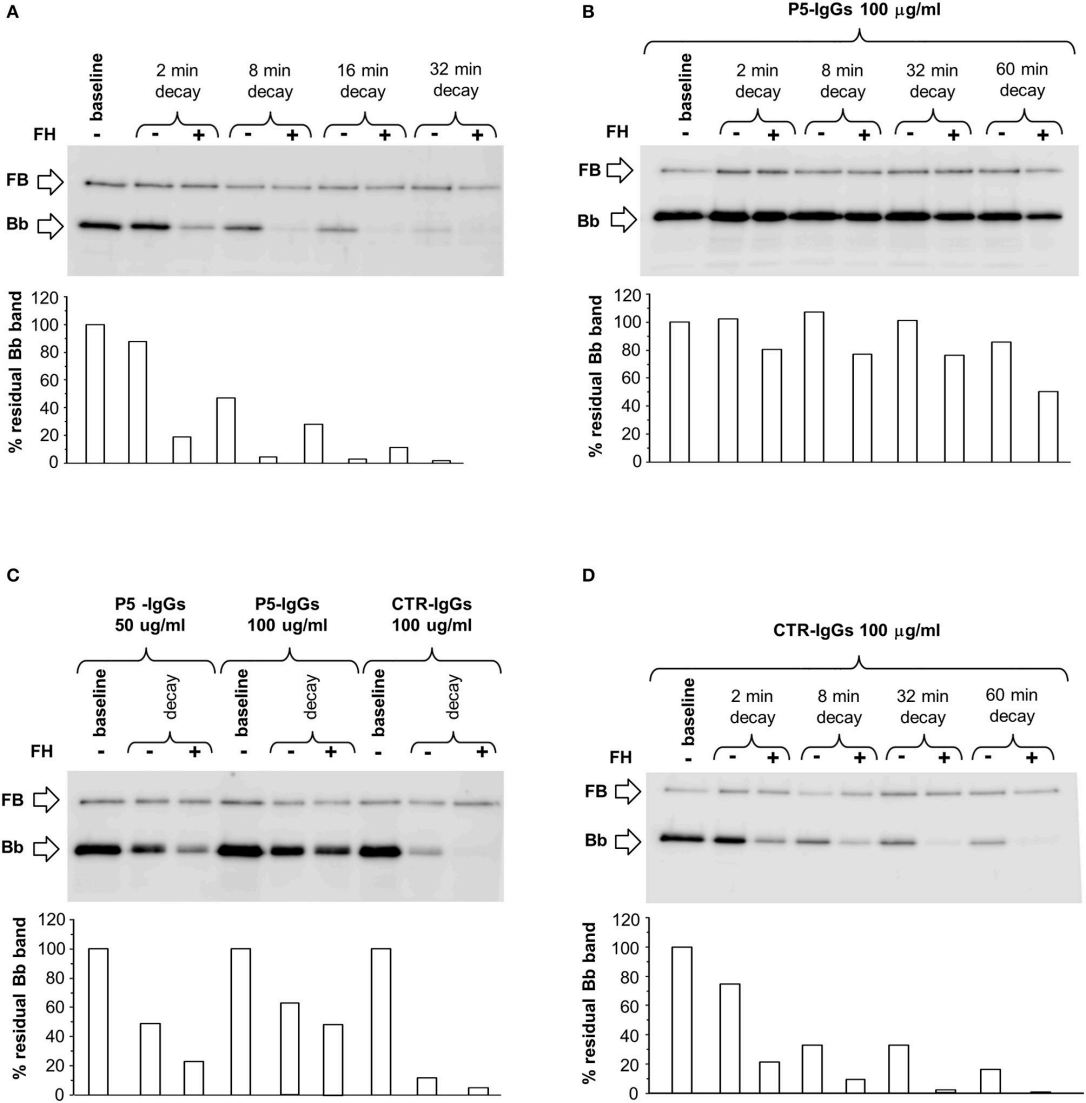
Spontaneous and FH-mediated decay of the C3bBb(Mg^2+^) C3 convertase in the absence or in the presence of IgGs from a DDD patient or a healthy control, by microplate/Western blot (WB) assay. **(A)** Time course of spontaneous and FH-mediated decay of C3bBb(Mg^2+^). The complexes were formed by incubating for 12 min at 25°C C3b-coated wells with 1,000 ng/ml FB, 10 ng/ml FD, and 10 mM MgCl_2_ (baseline). In additional wells after washing, the formed complexes were further incubated for 2, 8, 16, and 32 min in the absence (-) or in the presence (+) of 2,640 ng/ml FH. **(B)** C3bBb(Mg^2+^) was formed in the presence of IgGs from P5 patient with DDD during 12 min at 25°C (baseline). The complexes were allowed to decay for 2, 8, 32, and 60 min in the absence (–) or in the presence of 2,640 ng/ml FH (+). **(C)** C3bBb(Mg^2+^) was formed in the presence of IgGs (P5-IgGs, 50, and 100 μg/ml) from patient 5 or from an healthy subject (CTR-IgGs, 100 μg/ml) for 12 min at 25°C. Spontaneous or FH-mediated decay was monitored by further incubation for 32 min at 25°C with buffer alone (–) or added with 2,640 ng/ml FH (+), respectively. **(D)** C3bBb(Mg^2+^) was formed in the presence of IgGs from a healthy subject (CTR-IgGs) for 12 min at 25°C. The complexes were allowed to decay for 2, 8, 32, and 60 min in the absence (–) or in the presence of 2,640 ng/ml FH (+). The percentage of residual Bb band (visualized by an anti-FB antibody) was calculated as the ratio of the densities (in Pixel^2^) of each Bb band after decay and the density of the corresponding baseline Bb band before decay x 100 and results are reported in the bottom graphs. Results of a representative microplate/WB experiment of *n* = 3 for each sample are shown.

**Figure 2:**
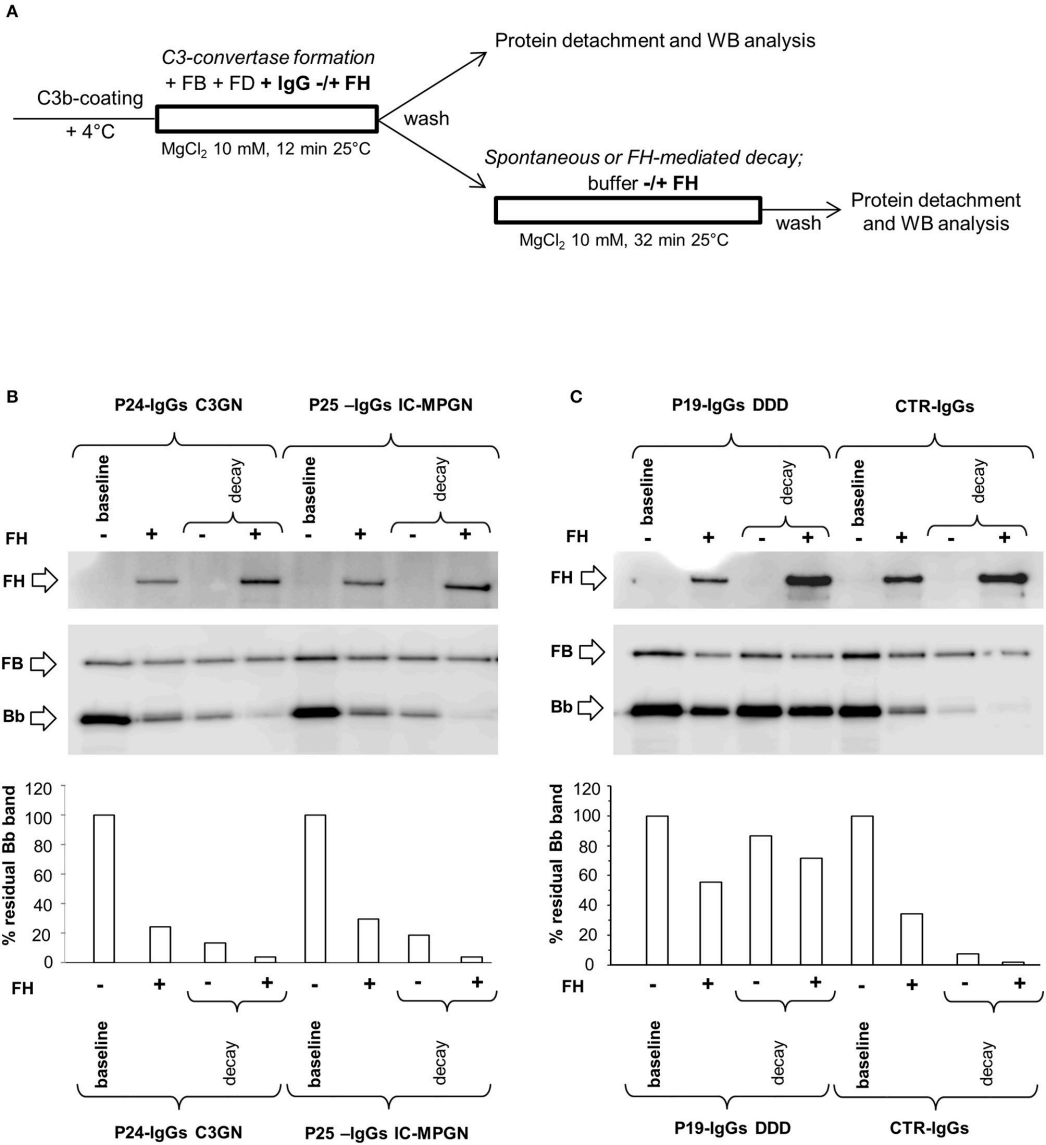
Effect of patient and control IgGs on decay of the C3bBb(Mg^2+^) C3 convertase by microplate/Western blot (WB) assay. **(A)** Experimental design (protocol 1) **(B-C)** Representative images of the assay. C3bBb(Mg^2+^) complexes were formed by incubating at 25°C for 12 min C3b-coated wells with 1,000 ng/ml FB, 10 ng/ml FD, 100 μg/ml IgGs purified from patients or healthy controls and 10 mM MgCl_2_ in the absence (–, baseline) or in the presence of 2,640 ng/ml FH. Spontaneous or FH-mediated decay of the complexes was monitored by further incubation of C3bBb(Mg^2+^) formed in the absence of FH for 32 min at 25°C with buffer alone (decay –) or 2,640 ng/ml FH (decay +), respectively. The percentage of residual Bb band (visualized by an anti-FB antibody) was calculated as the ratio of the densities (in Pixel^2^) of each Bb band after decay and the corresponding baseline Bb band density before decay x 100 and results are reported in the bottom graphs. The membranes were then incubated with an anti-FH antibody and FH band could be visualized at 150 KDa (top). Results of a representative microplate/WB experiment of *n* = 3 for each sample are shown.

Since C3NeFs are known to stabilize the C3 convertase against both spontaneous and/or FH-mediated decay (25, 31) we first studied the kinetics of spontaneous or accelerated C3 convertase decay. C3bBb(Mg^2+^) was allowed to form for 12 min on C3b-coated wells in the presence of FB and FD (baseline). In additional wells the formed complexes were subsequently incubated for different time intervals with buffer alone or with buffer containing FH at a physiological ratio with FB. As shown in Figure 1A, in the absence of FH, C3bBb(Mg^2+^) dissociated in a time-dependent manner. The decay of C3bBb(Mg^2+^) was strongly accelerated in the presence of FH. Indeed, no Bb band could be visualized after 8 min of decay with FH (Figure 1A), confirming that FH is very efficient in Bb displacement from C3b (21). In order to analyze the effect of C3NeFs on the stabilization of the C3bBb(Mg^2+^) C3 convertase complexes, the above experiments were repeated forming C3bBb(Mg^2+^) in the presence of 100 μg/ml IgGs purified from a C3G patient (P5) strongly positive for C3NeF or from a healthy subject (CTR) (protocol 1, Figure 2A). Results showed that IgGs from P5 greatly stabilized C3bBb(Mg^2+^) complexes by preventing spontaneous decay (Figure 1B), as documented by the intensity of the Bb band, which did not substantially change vs. baseline (before the decay). Similarly, P5 IgGs greatly prevented FH-mediated decay (Figure 1B). As shown in Figure 1C, the capability of P5 IgGs to stabilize C3bBb was dose-dependent. At variance, control IgGs did not affect either spontaneous or FH-accelerated C3bBb decay (Figures 1C,D).

Thereafter, 100 μg/ml IgGs from 26 patients who were C3NeF-positive in the hemolytic assay were tested for C3bBb stabilizing activity. IgG from 26 healthy donors were used to set the detection thresholds. Results were considered positive when the percentage of residual C3 convertase after spontaneous or FH-mediated decay was ≥ 37% or ≥12 % of baseline (> mean + 2SD of results from samples with control IgG, Supplementary Figures 2A,B), respectively.

Of the 26 tested patients' IgGs, 9 (35%) had C3 convertase-stabilizing activity (CSA) against both spontaneous (sCSA^+^) and FH-accelerated decay (FH-CSA^+^), one stabilized only spontaneous decay (P8), and one only the FH-mediated decay (P14) (Table 2). IgGs from all the C3G/IC-MPGN controls without C3NeF activity were sCSA^−^ and FH-CSA^−^ (Supplementary Figures 2A,B). The mean intra- and inter-assay CVs were 12.5 and 20.6% respectively for sCSA, and 12.4 and 18.4%, respectively for FH-CSA.

Because all components not bound to well surface were removed before the decay, following C3 convertase formation, the positivity in the above test indicates stable interaction between C3NeF IgGs and C3 convertase.

Figures 2B,C are representative images of WB analyses showing the effect of IgGs isolated from 3 patients (P19, DDD; P24, C3GN, and P25 IC-MPGN) and one healthy control on C3 convertase stabilization against the spontaneous and accelerated decay. P19 IgG strongly stabilized C3bBb(Mg^2+^) complexes by preventing both spontaneous and FH-mediated decay. In contrast, P24 and P25 IgGs failed to prevent spontaneous and accelerated decay.

We observed a trend toward a higher prevalence of sCSA^+^ and FH-CSA^+^ C3NeF IgGs among patients with DDD than in patients in the other histology groups but the difference did not reach statistical significance (Table 3A).

**Table 3:** Prevalence of patients with C3NeF IgGs with C3 convertase stabilizing activity (CSA) against spontaneous (s) or FH-mediated (FH) decay in the absence (sCSA, FH-CSA) or in the presence of properdin, (sPCSA, FH-PCSA) among histology groups or clusters.

	**C3GN**	**DDD**	**IC-MPGN**	***p***	**Cluster 1**	**Cluster 2**	**Cluster 3**	***p***
sCSA^+^	25%	67%	23%	0.115	22%	13%	78%	**0.017**
FH-CSA^+^	25%	67%	23%	0.115	22%	13%	78%	**0.017**
sPCSA^+^	100%	100%	92%	1.000	100%	88%	100%	0.571
FH-PCSA^+^	100%	78%	85%	1.000	89%	75%	89%	0.653

*Bold characters indicate statistically significant values*.

**Table 3B:** Prevalence of patients with C3NeF IgGs with properdin (P) independent (CSA+/PCSA+) or dependent (CSA–/PCSA+) C3 convertase stabilizing activity against spontaneous (s) or FH-mediated (FH) decay among histology groups or clusters.

		**C3GN**	**DDD**	**IC-MPGN**	***p***	**Cluster 1**	**Cluster 2**	**Cluster 3**	***p***
Spontaneous	sCSA^−^/sPCSA^−^	0%	0%	8%	1.000	0%	13%	0%	0.308
	sCSA^−^/sPCSA^+^	75%	33%	69%	0.239	78%	75%	22%	**0.040**
	sCSA^+^/sPCSA^+^	25%	67%	23%	0.115	22%	13%	78%	**0.017**
FH-mediated	FH-CSA^−^/FH-PCSA^−^	0%	22%	15%	1,000	11%	25%	11%	0.653
	FH-CSA^−^/FH-PCSA^+^	75%	11%	62%	**0.026**	67%	63%	11%	**0.042**
	FH-CSA^+^/FH-PCSA^+^	25%	67%	23%	0.115	22%	13%	78%	**0.017**

*Bold characters indicate statistically significant values*.

A clear-cut difference in the C3 convertase-stabilizing activity of IgGs was found when we grouped the patients according to recently reported cluster analysis (15). Nine patients (the 4 with C3GN, 1 with DDD and 4 with IC-MPGN) fell into cluster 1 and were characterized by low serum C3 and very high plasma sC5b-9 at onset, and subendothelial and frequently subepithelial and mesangial deposits (Tables 1, 4). Eight patients (all with IC-MPGN) were grouped in cluster 2, and showed low serum C3, high plasma sC5b-9, prevalent subendothelial deposits and stronger glomerular staining of IgG and C1q (*P* < 0.01) than patients in clusters 1 and 3 (Tables 1, 4). Finally, the 9 patients of cluster 3 (8 with DDD and 1 with IC-MPGN) had low serum C3 and normal plasma sC5b-9 levels (*P* < 0.01 vs. clusters 1 and 2). Cluster 3 patients, including the one with a diagnosis of IC-MPGN, were characterized by intramembranous highly electron-dense deposits (Tables 1, 4).

**Table 4:** Comparison of clinical, and biochemical parameters at onset and histology features between clusters.

**Feature**	**Cluster 1 (*n* = 9)**	**Cluster 2 (*n* = 8)**	**Cluster 3 (*n* = 9)**	***P***
Gender (male %)	56%	38%	67%	0.547
Age at onset (y)	13.6 (±3.55)	12.6 (±8.1)	11.6 (±5.72)	0.777
Serum C3 (mg/dl)	27 (±16.8)	22.2 (±17.2)	20.9 (±18)	0.739
Plasma sC5b9 (ng/ml)	1800 (±1150)*	1820 (±1210)*	296 (±118)	**0.003**
u-Protein (g/24h)	3.3 (±3.1)	5.7 (±4.9)	1.7 (±1.9)	0.099
s-Creatinine (mg/dl)	0.68 (±0.18)	0.71 (±0.19)	0.59 (±0.26)	0.426
C3 at IF	2.94 (±0.17)	2.94 (±0.18)	2.72 (±0.36)	0.137
IgG at IF	0.67 (±1.12)	2.50 (±0.54)°	0.28 (±0.44)	**9.6E-06**
C1q at IF	0.50 (±0.71)	1.50 (±0.89)°	0.278 (±0.44)	**0.003**
Mesangial deposits at EM	67%	38%	56%	0.547
Subepithelial deposits at EM	78%*	38%	11%	**0.016**
Subendothelial deposits at EM	100%*	100%*	0%	**6.4E-07**
Intramembr. highly dense deposits at EM	11%	0%	100%#	**6.4E-06**

*EM, electron microscopy; u-protein, proteinuria; s-Creatinine, serum creatinine*.

Data are expressed as mean ± SD or as % of positive patients.

* Significantly different vs. cluster 3.

° Significantly different vs. clusters 1 and 3.

# Significantly different vs. clusters 1 and 2.

*Bold characters indicate statistically significant values*.

**Figure 3:**
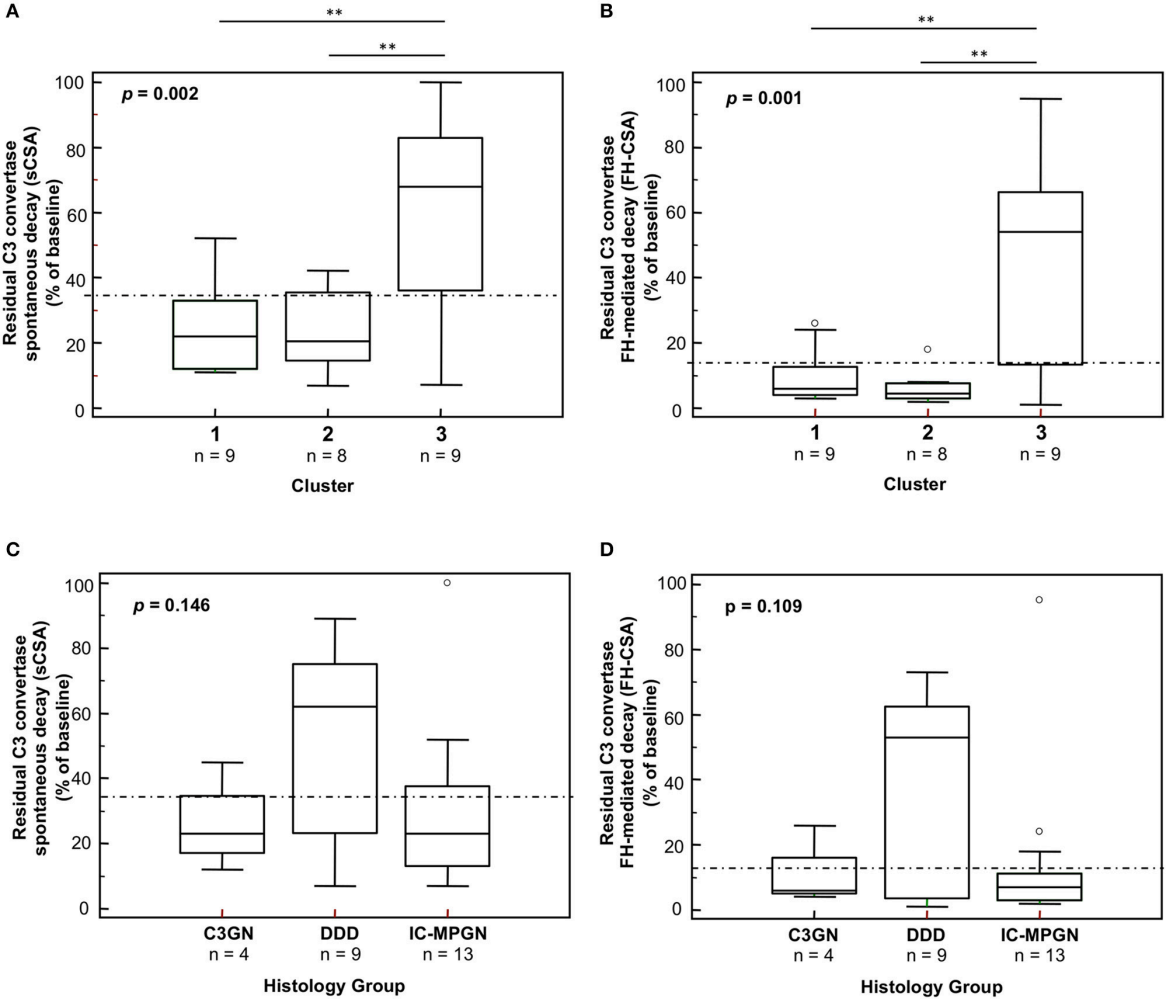
Distribution of the C3NeF IgG C3 convertase-stabilizing activities in the absence of properdin according to the clusters and the histology groups. Box plots show significantly higher C3 convertase stabilizing activities against spontaneous (sCSA) and FH-mediated (FH-CSA) decay (protocol 1) in patients of cluster 3 than cluster 1 and 2 patients **(A,B)**. No significance difference was observed among histology groups **(C,D)**. The boxes represent the values from the 25th to 75th percentiles. The horizontal bars are the medians. Vertical lines are the 95% confidence intervals. Empty circles are values outside the 95% confidence intervals. The dashed horizontal lines show the limit of positive values (set at >mean+2SD of results with control IgGs from 26 healthy subjects). **P < 0.01.

A significantly higher prevalence of patients who fell within cluster 3 had C3NeF IgGs that stabilized the C3 convertase against spontaneous (sCSA^+^) and FH-accelerated (FH-CSA^+^) than did patients from clusters 1 and 2 (Table 3A). Levels of sCSA and FH-CSA activities of C3NeF IgGs were significantly higher in cluster 3 than in clusters 1 and 2 (Figures 3A,B), while no significant difference in C3NeF IgGs activities was observed between histology groups (Figures 3C,D).

C3 convertase-stabilizing activity values of C3NeF IgGs negatively correlated with plasma sC5b-9 levels (r = −0.49, *P* = 0.01 for both sCSA and FH-CSA activities) measured at the time of IgG isolation, whereas no correlation was found between C3NeF activity measured by hemolytic assay and plasma sC5b-9 (r = 0.15, *P* = 0.48). Neither C3 convertase-stabilizing activities nor C3NeF activity by hemolytic assay correlated with C3d/C3 ratios (sCSA, r = −032, *P* = 0.14; FH-CSA, r = −0.27, *P* = 0.21; hemolytic assay, r = 0.01, *P* = 0.98). C3 convertase-stabilizing activity values did not correlate with results of the hemolytic assays (sCSA, r = 0.27, *P* = 0.19; FH-CSA, r = 0.31, *P* = 0.12).

### Effect of C3NeF on preformed C3 convertase

**Figure 4:**
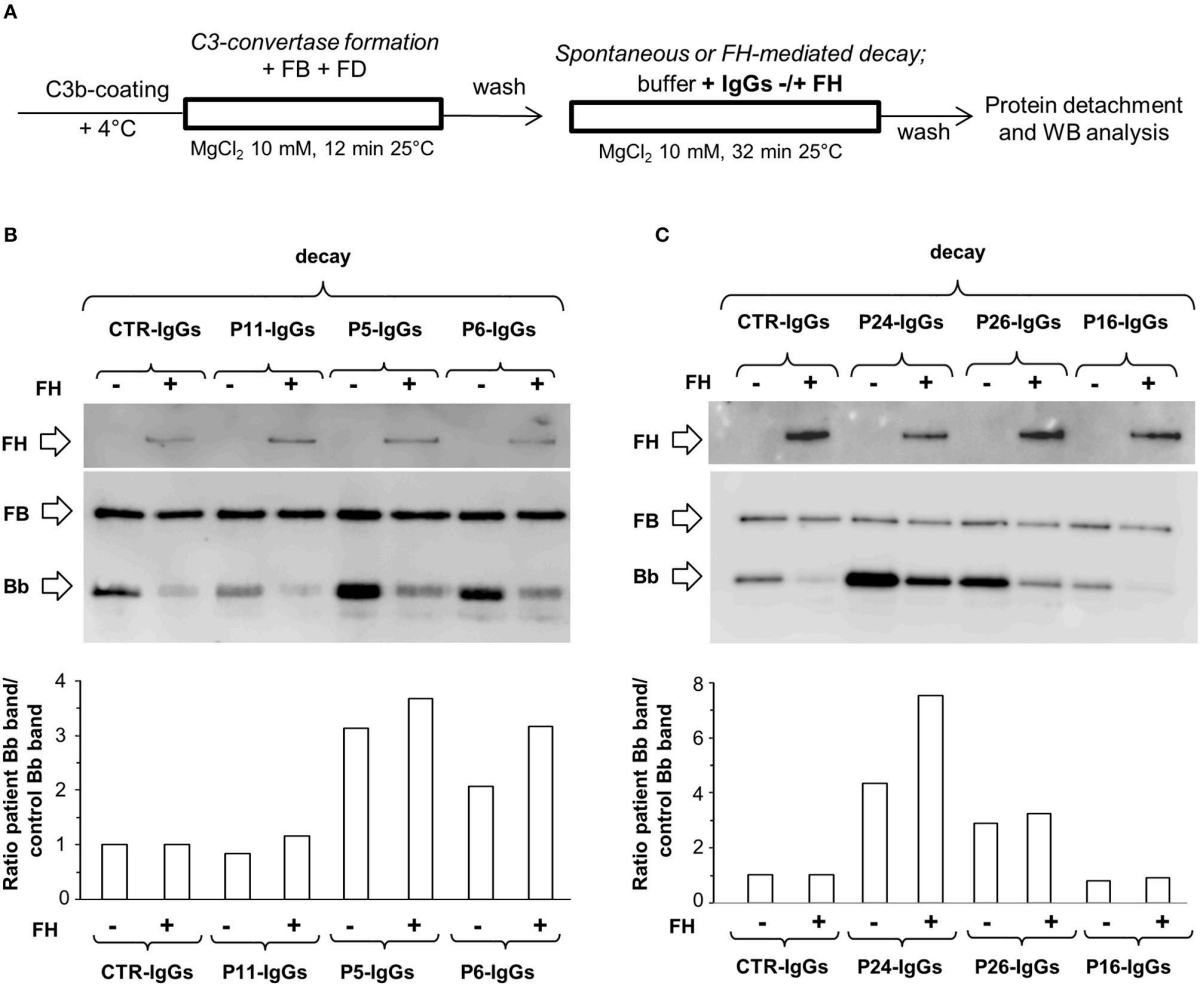
Effect of patient and control IgGs on preformed C3bBb(Mg^2+^) C3 convertase by microplate/Western blot (WB) assay. **(A)** Experimental design (protocol 2). **(B,C)** Representative images of the assay. C3bBb(Mg^2+^) complexes were formed by incubating at 25°C for 12 min C3b-coated wells with 1,000 ng/ml FB, 10 ng/ml FD, and 10 mM MgCl_2_. The complexes formed were then incubated in the presence of 100 μg/ml IgGs from patients or healthy controls without (spontaneous decay, decay –) or with (FH-mediated decay, decay +) 2,640 ng/ml FH. The C3NeF IgG C3 convertase-stabilizing activity was quantified as the ratio of the densities (in Pixel^2^) of Bb bands (visualized by an anti-FB antibody) after decay in the presence of patient IgGs/ Bb band after decay in the presence of control IgGs (ratio patient Bb band/control Bb band) and results are reported in the bottom graphs. The membranes were then incubated with an anti-FH antibody and FH band could be visualized at 150 KDa (top). Results of a representative microplate/WB experiment of *n* = 3 for each sample are shown.

In order to evaluate the ability of C3NeF IgGs to stabilize preformed C3 convertase, we modified the above assays by adding the IgGs after C3 convertase formation, only during the decay step (protocol 2, Figure 4A). Results were considered positive when the ratio of residual convertase in the sample with C3NeF IgGs vs. residual convertase in the sample with control IgGs run in parallel was >1.9 or >2.3 for spontaneous or FH-mediated decay, respectively (ratios between the mean + 2SD and the mean of residual Bb band in the reactions with IgGs from 26 healthy subjects). Of the 26 tested patients' C3NeF IgGs, all those that prevented both spontaneous and FH-mediated decay when added during C3 convertase assembly in protocol 1, also stabilized preformed C3 convertase in protocol 2.

Notably, IgGs from P24 stabilized C3 convertase against both spontaneous and FH-mediated decay only when added during decay (protocol 2, Figure 4A and Table 2), suggesting that these IgGs interacted weakly with C3 convertase. The IgGs from the other C3NeF^+^ patients and from all the C3G/IC-MPGN controls without C3NeF activity by hemolytic assay (Supplementary Figures 2E,F) were negative in protocol 2. Representative images of the results of the assay on preformed C3 convertase are shown in Figures 4B,C.

### Effect of properdin on C3NeF stabilizing activity on C3 convertase

Previous studies indicated that some C3NeFs need properdin to exert their stabilizing activity on C3 convertase (26, 28, 31, 38, 39).

**Figure 5:**
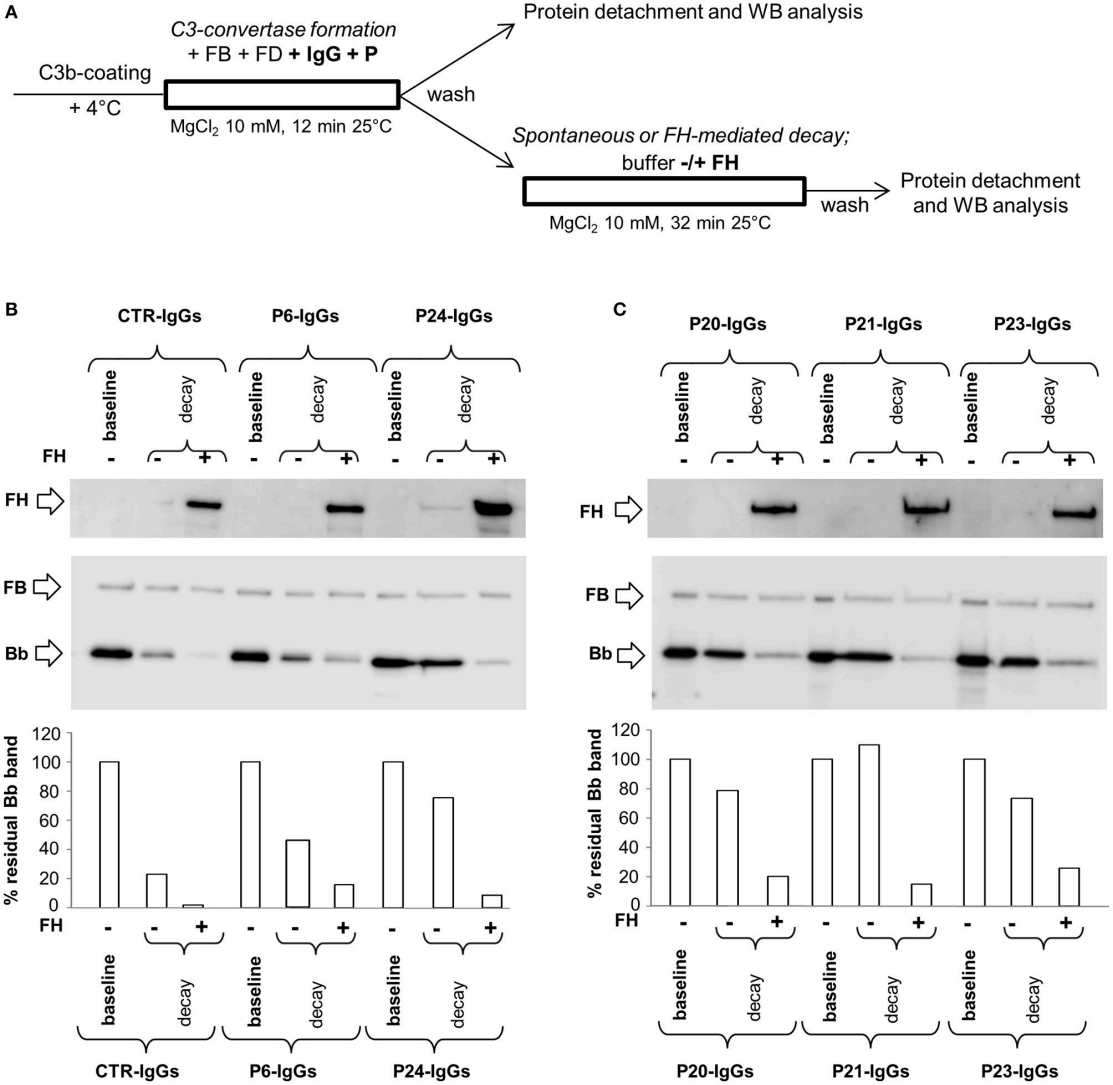
Effect of properdin on C3 convertase-stabilizing activity of C3NeF IgGs. **(A)** Experimental design (protocol 3). **(B,C)** Representative images of the assay. C3bBb(Mg^2+^) complexes were formed by incubating at 25°C for 12 min C3b-coated wells with 1,000 ng/ml FB, 10 ng/ml FD, 100 μg/ml IgGs from patients or healthy controls and 10 mM MgCl_2_ in the presence of 500 ng/ml Properdin (P) (baseline). In additional wells after washing, spontaneous or FH-mediated decay of the formed complexes was monitored by further incubation for 32 min at 25°C with buffer alone (decay –) or buffer added with 2,640 ng/ml FH (decay +), respectively. The percentage of residual Bb band was calculated as the ratio of the densities (in Pixel^2^) of each Bb band (visualized by an anti-FB antibody) after decay and the corresponding baseline Bb band density before decay x 100, and results are reported in the bottom graphs. The membranes were then incubated with an anti-FH antibody and FH band could be visualized at 150 KDa (top). Results of a representative microplate/WB experiment of *n* = 3 for each sample are shown.

In order to investigate the possible synergic effect of properdin (P) with C3NeF IgGs in stabilizing C3 convertase, P (at a physiological molar ratio with FB) was added during C3 convertase formation together with FB, FD and IgGs in C3b-coated wells for 12 min (protocol 3, Figure 5A). Thereafter, the C3bBbP complexes were allowed to dissociate for 32 min in the presence or in the absence of FH. Results were considered positive when the percentage of residual convertase after spontaneous or FH-mediated decay was ≥ 42 or ≥10 %, respectively (> mean + 2SD of results from samples with IgGs isolated from 26 healthy donors, Supplementary Figures 2C,D). We found that of the 17 C3NeF IgGs that did not stabilize the C3 convertase in the assay without properdin (protocol 1), 13 had stabilizing activity both on spontaneous and FH-accelerated decay of C3bBbP C3 convertase in the presence of properdin (sPCSA^+^ and FH-PCSA^+^), three (P2, P9, and P16) prevented C3bBbP spontaneous decay only (sPCSA^+^ and FH-PCSA^−^), while one (from P29) had no effect. The latter patient carries a likely pathogenetic variant in CFB and showed low C3NeF activity in the hemolytic assay (Table 2). Finally, the C3NeF IgGs that stabilized C3 convertase in the absence of properdin also stabilized the C3 convertase in the assay with properdin (Table 2 and representative images in Figures 5B,C). IgGs from all the C3G/IC-MPGN controls without C3NeFs were negative also in the C3 convertase stabilizing assay with properdin (Supplementary Figures 2C,D). The mean intra- and inter-assay CVs were 8.2 and 18.6% respectively for sPCSA, and 6.7 and 23%, respectively for FH-PCSA.

C3NeF IgGs with properdin-dependent C3 convertase-stabilizing activity (sCSA^−^/sPCSA^+^ and/or FH-CSA^−^/FH-PCSA^+^) were more frequently found among patients with C3GN (3 of 4 for both) or IC-MPGN (sCSA^−^/sPCSA^+^: 9 of 13 and FH-CSA^−^/FH-PCSA^+^: 8 of 13 patients) than among patients with DDD (sCSA^−^/sPCSA^+^: 3 of 9 and FH-CSA^−^/FH-PCSA^+^: 1 of 9 patients) (Table 3B), however, difference reached statistical significance only for stabilization against FH-mediated decay.

The difference in properdin-dependence of C3NeFs was more evident between clusters: patients who fell within clusters 1 and 2 were more likely to have properdin-dependent C3NeFs (cluster 1: sCSA^−^/sPCSA^+^: 7 of 9 and FH-CSA^−^/FH-PCSA^+^: 6 of 9 patients; cluster 2: sCSA^−^/sPCSA^+^: 6 of 8 and FH-CSA^−^/FH-PCSA^+^ 5 of 8 patients) than patients in cluster 3 (sCSA^−^/sPCSA^+^: 2 of 9 and FH-CSA^−^/FH-PCSA^+^: 1 of 9 patients) (Table 3B).

**Figure 6:**
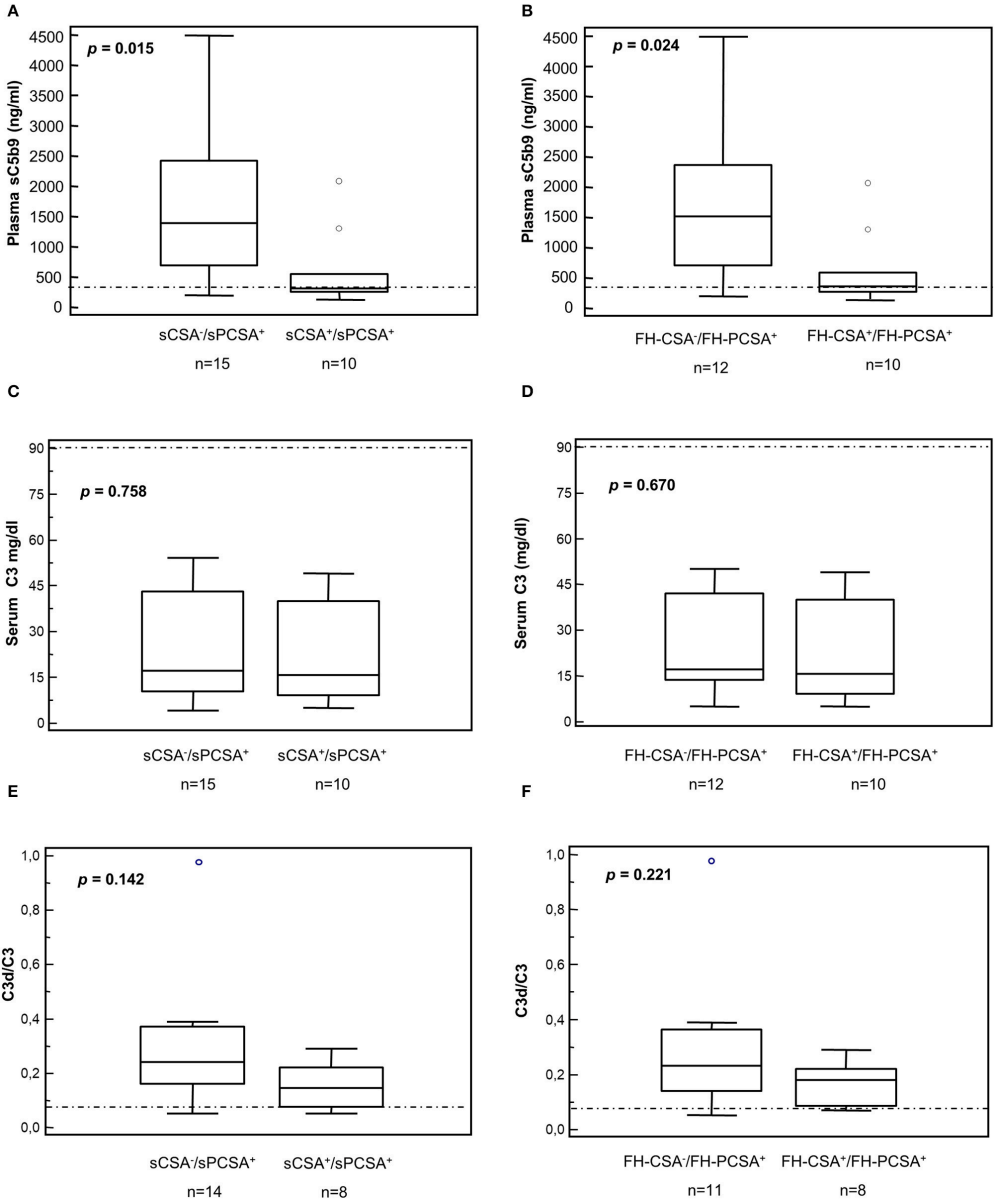
Distribution of serum C3 and plasma sC5b-9 levels and plasma C3d/C3 ratios in patients according to results of C3 convertase stabilizing-activities in the absence or in the presence of properdin. **(A,B)** Plasma sC5b-9 levels. Box plots show that patients carrying C3NeFs with properdin-dependent C3 convertase-stabilizing activity against spontaneous (**A**, sCSA^−^/sPCSA^+^) and/or FH-mediated (**B**, FH-CSA^−^/FH-PCSA^+^) decay had higher plasma sC5b-9 levels than patients carrying C3NeF with properdin-independent activities (**A,B**, sCSA^+^/sPCSA^+^ and/or FH-CSA^+^/FH-PCSA^+^). No significant difference was found in C3 levels and in C3d/C3 ratios **(C–F)**. The boxes represent the values from the 25 to 75th percentiles. The horizontal bars are the medians. Vertical lines are the 95% confidence intervals. Empty circles are values outside the 95% confidence intervals. The horizontal dashed lines show the upper limits of normal range of plasma sC5b-9 **(A,B)** and of the C3d/C3 ratio **(E,F)**, and the lower limit of serum C3 **(C,D)** in healthy subjects.

Patients carrying C3NeFs with properdin-dependent C3 convertase-stabilizing activity (sCSA^−^/sPCSA^+^ or FH-CSA^−^/FH-PCSA^+^) had higher plasma sC5b-9 levels than patients carrying C3NeFs that were also active in the assay without properdin (sCSA^+^/sPCSA^+^ or FH-CSA^+^/FH-PCSA^+^, Figures 6A,B, Table 5 and Supplementary Table 1). C3d/C3 ratios were significantly higher (*P* < 0.01) than in healthy subjects (0.05 ± 0.02, *n* = 15) in the overall group of C3NeF^+^ patients (0.24 ± 0.20, *n* = 23) as well as in the subgroups of patients with properdin-dependent or properdin-independent C3NeFs (Figures 6E,F and Table 2). C3 and C3d/C3 values did not significantly differ between patients with properdin dependent and properdin independent C3NeFs (Figures 6C–F).

**Table 5:** Clinical features, complement assessment, genetic screening and histologic features in patients classified according to the type of C3 convertase stabilizing activity (spontaneous decay).

**Variable**	**sCSA^−^/sPCSA^−^**	**sCSA^−^/sPCSA^+^**	**sPCSA^+^/sPCSA^+^**	**Overall p-value**	**^−^/+ vs. +/+ *p*-value**
N	1	15	10		
Gender (% males)	0%	40%	80%	0.068	0.099
**Data at onset**					
Age (y) - Mean (SD)	5.3	12.8 (±5.1)	13 (±6.8)	0.454	0.953
Microhematuria	100%	87%	89%	1.00	1.00
Gross hematuria	0%	20%	60%	0.087	0.087
Proteinuria	0%	100%	90%	**0.031**	0.400
Nephrotic syndrome	0%	53%	30%	0.526	0.414
Renal impairment	0%	7%	0%	1.00	1.00
Familiarity for nephropathy	0%	13%	0%	0.538	0.500
Serum C3 (mg/dl)	36	24.5 (±17.2)	21.6 (±16.5)	0.705	0.682
Serum C4 (mg/dl)	15	17.3 (±8.8)	23.8 (±9)	0.190	0.083
Plasma sC5b-9 (ng/ml)	1600	1731 (±1277)	581 (±623)	**0.047**	**0.015**
Low serum C3 and normal serum C4	100%	73%	90%	0.689	0.615
C3NeF positive	100%	100%	100%	1.00	1.00
LPV carriers	100%	20%	20%	0.270	1.00
**Data during follow-up**					
Nephrotic syndrome	0%	87%	40%	**0.016**	**0.028**
High blood pressure	0%	47%	56%	1.00	1.00
Chronic kidney disease	0%	20%	40%	0.546	0.378
ESRD	0%	7%	10%	1.00	1.00
**Histological features**					
Time Onset to Biopsy (year)	5	1 (±1.8)	1.3 (±2.1)	0.145	0.693
**Light microscopy**					
Sclerotic glomeruli	8%	0% (±2%)	0% (±1%)	**6.0** × **10**^−4^	0.809
Crescents	0%	2% (±6%)	15% (±30%)	0.277	0.123
Degree of mesangial proliferation*	1	1.3 (±1.3)	1.6 (±0.7)	0.839	0.642
Degree of endocapillary proliferation*	0	0.9 (±0.9)	1.1 (±1.1)	0.556	0.665
Degree of interstitial inflammation*	0	0.8 (±0.8)	0.6 (±0.5)	0.485	0.456
Degree of interstitial fibrosis*	1	0.3 (±0.6)	0.2 (±0.4)	0.424	0.791
Degree of arteriolar sclerosis*	0	0.1 (±0.3)	0.1 (±0.3)	0.913	0.755
**Immunofluorescence**					
C3*	3	2.8 (±0.4)	2.8 (±0.4)	0.741	0.604
IgA*	2	0.4 (±0.6)	0 (±0)	**0.001**	**0.040**
IgG*	3	1.5 (±1.3)	0.7 (±1)	0.125	0.142
IgM*	2	1.2 (±0.9)	0.7 (±0.7)	0.230	0.186
C1q*	0	1.1 (±1)	0.6 (±0.9)	0.352	0.262
Fibrinogen*	3	0.4 (±0.6)	0 (±0)	**4.4** × **10**^−5^	0.076
**Electron microscopy**					
Mesangial deposits	0%	64%	30%	0.157	0.098
Subepithelial deposits	100%	36%	30%	0.494	1.00
Subepithelial hump-like deposits	0%	17%	20%	1.00	1.00
Subendothelial deposits	100%	86%	30%	**0.009**	**0.010**
Intramembranous granular deposits	0%	57%	30%	0.227	0.240
Intramembranous highly electron-dense deposits	0%	21%	70%	**0.034**	**0.035**

* *Degree of mesangial proliferation, endocapillary proliferation, interstitial inflammation, interstitial fibrosis, and arteriolar sclerosis, as well as IF findings were graded using a scale of 0 to 3, including 0, trace (0.5+), 1+, 2+, and 3+. Quantitative variables are expressed as mean (±S.D.) unless otherwise specified. Serum C3, reference 90-180 mg/dl; serum C4, reference 10-40 mg/dl; plasma sC5b-9, reference ≤ 400 ng/ml. LPV, Likely pathogenetic variants. sCSA-/sPCSA-, patients without C3 convertase stabilizing activity; sCSA-/sPCSA+, patients with properdin-dependent C3 convertase stabilizing activity; sPCSA+/sPCSA+, patients with properdin-independent C3 convertase stabilizing activity. Bold characters indicate statistically significant values*.

**Figure 7:**
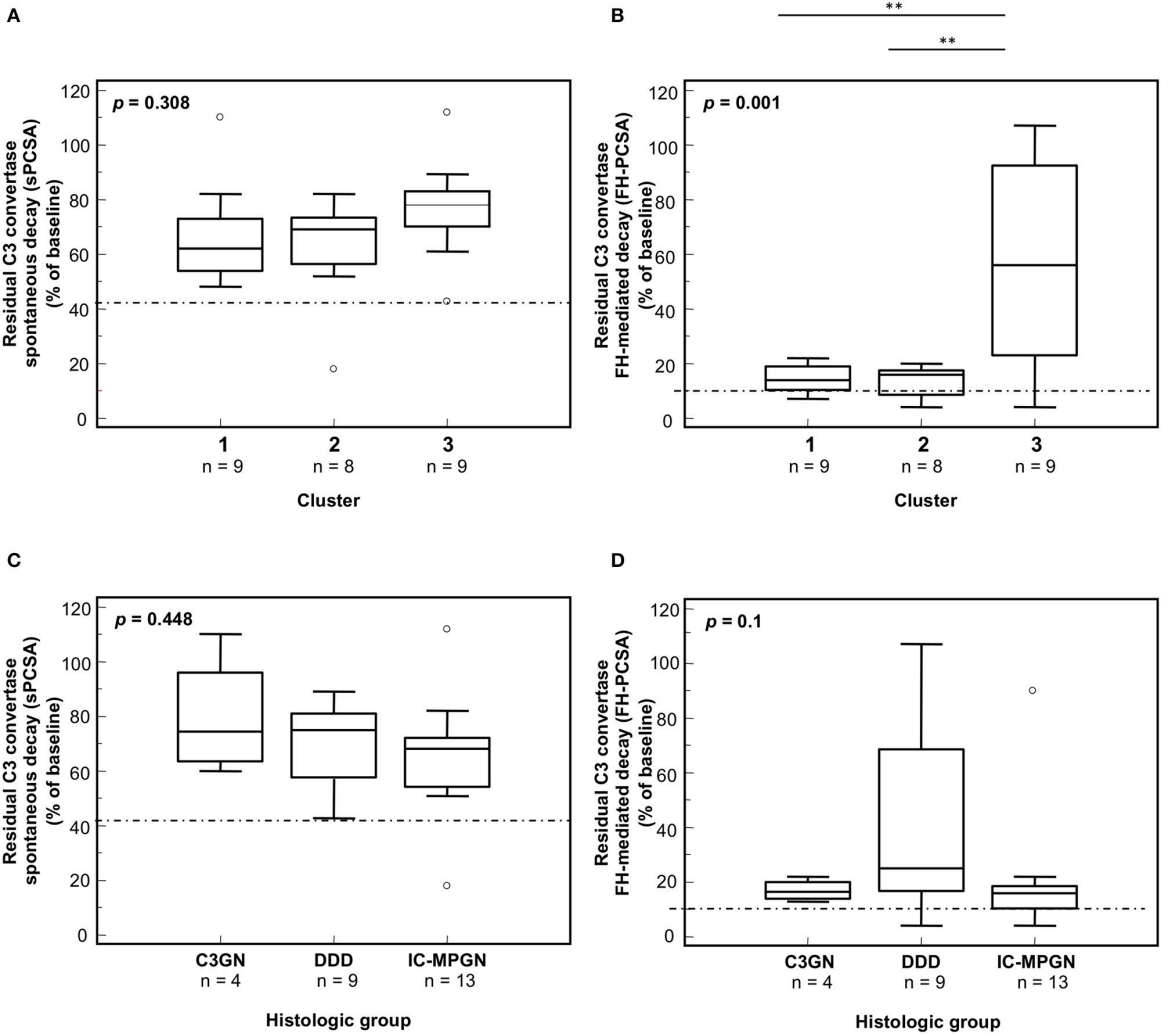
Distribution of the C3NeF IgG C3 convertase-stabilizing activities in the presence of properdin according to the clusters and the histology groups. Box plots show that C3 convertase-stabilizing activity against spontaneous (sPCSA) **(A,C)** decay did not differ among clusters or histology groups. C3 convertase-stabilizing activity against FH-mediated decay (FH-PCSA) **(B,D)** was significantly higher in patients of cluster 3 than in patients of clusters 1 and 2. The boxes represent the values from the 25 to 75th percentiles. The horizontal bars are the medians. Vertical lines are the 95% confidence intervals. Empty circles are values outside the 95% confidence intervals. The dashed horizontal line shows the limit of positive values (set at >mean+2SD of results with control IgGs from 26 healthy subjects). **P < 0.01.

In the presence of properdin, levels of spontaneous C3 convertase stabilizing activity (sPCSA) of C3NeF IgGs did not differ between histology groups or clusters (Figures 7A,C), while stabilizing activity against FH-mediated decay (FH-PCSA) was significantly higher for C3NeF IgGs from cluster 3 vs. clusters 1 and 2 (Figures 7B,D).

### Clinical and histology parameters in C3G/IC-MPGN patients according to type of C3 convertase stabilizing activity

**Figure 8:**
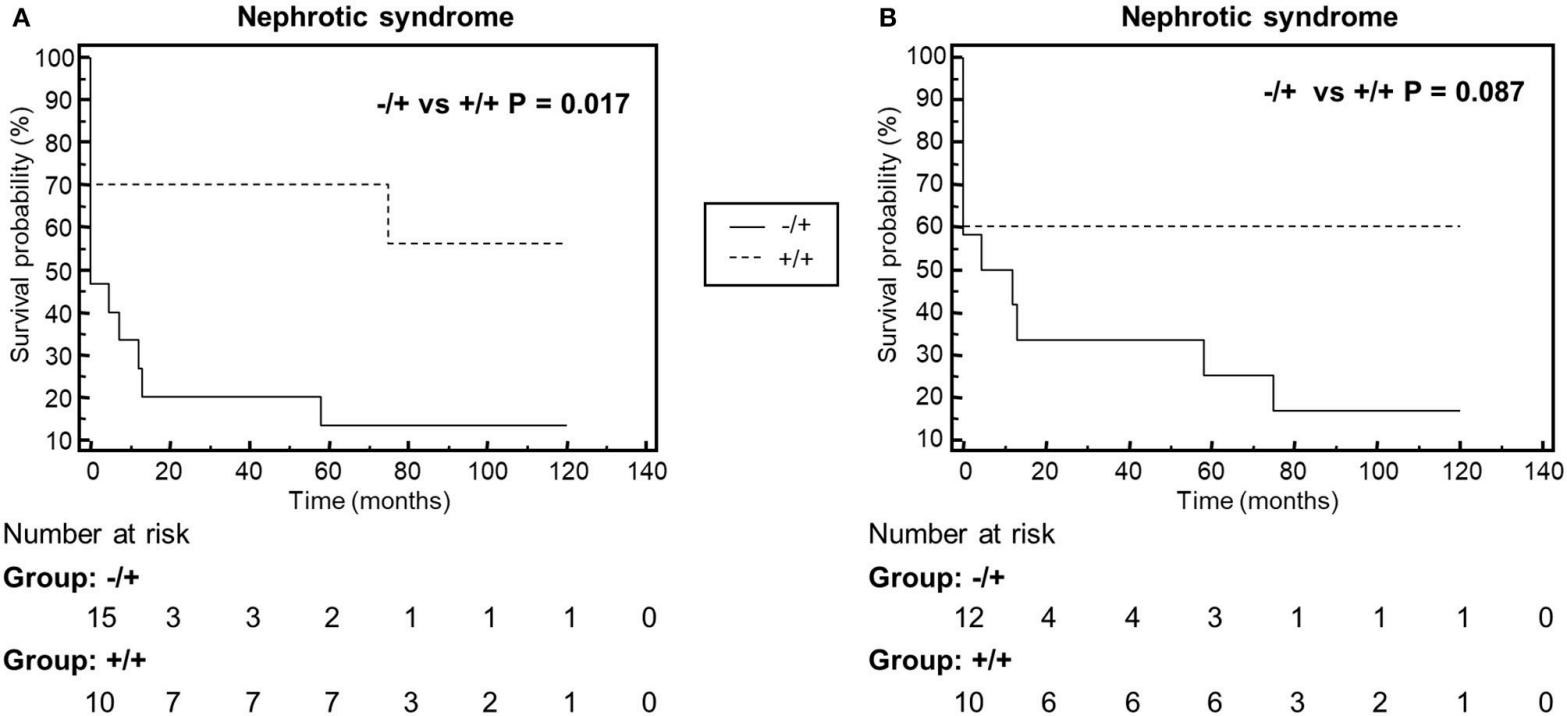
Kaplan-Meyer analysis of the event nephrotic syndrome during the disease course. The analysis shows that patients with properdin dependent C3 convertase stabilizing activity against spontaneous (**A**, Group ^−^/+: sCSA^−^/sPCSA^+^) or FH-mediated (**B**, Group ^−^/+: FH^−^CSA^−^/FH^−^PCSA^+^) decay, have higher risk to manifest nephrotic syndrome than patients with properdin-independent activities (**A**, Group +/+: sCSA^+^/sPCSA^+^; **B**, Group +/+: FH^+^CSA^+^/FH^+^PCSA^+^.

We compared clinical parameters at onset and during follow-up and bioptic findings in the groups of patients with properdin-dependent vs. patients with properdin-independent C3 convertase-stabilizing activity. Patients with properdin-dependent activity (sCSA^−^/sPCSA^+^ or FH-CSA^−^/FH-PCSA^+^) were more likely to have subendothelial deposits in kidney biopsy, and to develop nephrotic syndrome during disease course, while intramembranous highly electron dense deposits were more prevalent in patients with properdin-independent activity (sCSA^+^/sPCSA^+^ or FH-CSA^+^/FH-PCSA^+^) (Figure 8, Table 5 and Supplementary Table 1).

### C3NeFs do not prevent FH interaction with C3 convertase

To investigate the mechanisms by which C3NeFs stabilize the C3 convertase, we then evaluated whether IgGs from patients with C3G/IC-MPGN prevent the interaction of FH with C3 convertase. For this purpose, WB membranes of products recovered after the decay of C3bBb(Mg^2+^) complexes with C3NeF IgGs or control IgGs added either only during C3bBb(Mg^2+^) formation in the absence or in the presence of properdin (protocols 1 and 3) or only during decay (protocol 2), were labeled with an anti-FH antibody. As shown in the top of the representative WB images of Figures 2B,C, **4B,C**, **5B,C**, FH band could be clearly seen in all samples with no relevant difference between C3NeF IgGs and control IgGs. These results were confirmed with all the C3NeF IgGs of the 26 tested patients.

Altogether, the above results suggest that (1) C3NeF IgGs added during C3 convertase formation did not affect the interaction of FH with C3b and with nascent C3 convertase; (2) C3NeF IgGs added during decay did not compete with FH for the interaction with the C3 convertase.

### Effect of C3NeF on C3 convertase formation

To evaluate whether C3NeFs had an effect on C3bBb(Mg^2+^) formation in the presence or in the absence of FH, we analyzed the density of the Bb bands at the baseline of protocol 1 experiments, in which the IgGs were added during the C3bBb formation. In the reactions with control IgGs the amount of C3bBb(Mg^2+^) formed in the presence of FH was lower compared to C3bBb(Mg^2+^) formed in the absence of FH (ratio Bb band + FH /Bb band without FH: 0.32 ± 0.08). FH-mediated inhibitory effect on C3bBb(Mg^2+^) formation was significantly attenuated in the presence of C3NeF IgGs that in protocol 1 stabilized C3 convertase against FH-accelerated decay (FH-CSA^+^ IgGs: ratio Bb band +FH /Bb band without FH: 0.58 ± 0.19, *p* < 0.05 vs. control IgGs).

At variance, FH-CSA^−^ C3NeF IgGs did not affect FH-mediated inhibitory effect on C3bBb(Mg^2+^) formation (ratio Bb band + FH /Bb band without FH: 0.32 ± 0.07).

Representative images are shown in Figures 2B,C.

### Effect of C3NeF on C3bB C3 proconvertase

**Figure 9:**
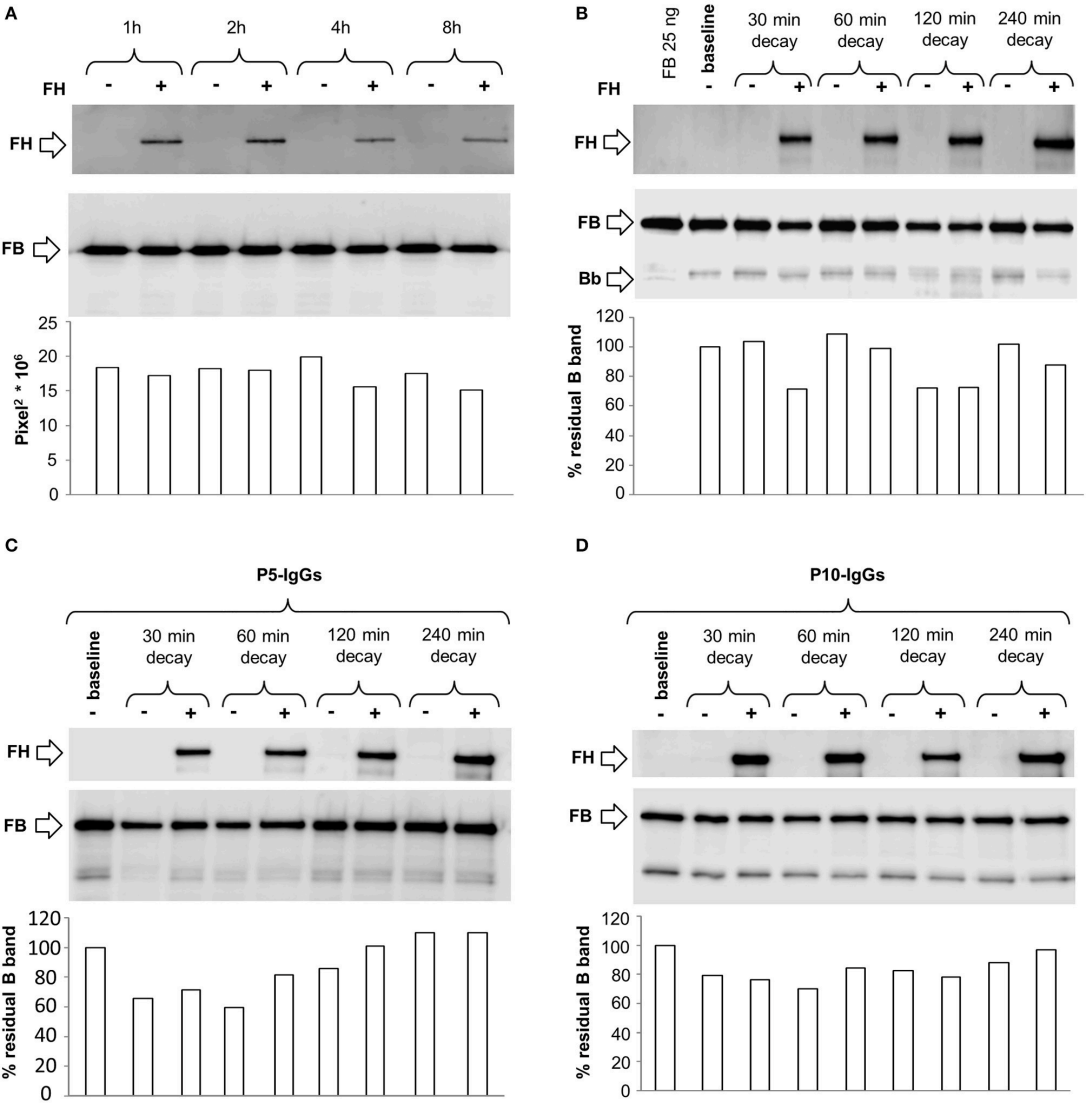
Formation and decay of C3bB(Mn^2+^) C3 proconvertase in the absence or in the presence of C3NeF IgGs, by microplate/WB assay. **(A)** Time course of C3bB(Mn^2+^) C3 proconvertase formation. The complexes were obtained incubating C3b-coated wells at 37°C for 1, 2, 4, and 8 h with 1,000 ng/ml FB and 2 mM MnCl_2_ in the absence (-) or in the presence (+) of 2,640 ng/ml FH. The amount of C3bB formed was calculated as the density of B band (93 KDa), and reported in the bottom graph as Pixel^2^* 10^6^. **(B–D)** Time course of C3bB(Mn^2+^) C3 proconvertase spontaneous and FH-mediated decay. C3bB(Mn^2+^) complexes formed in 2 h at 37°C in the absence **(B)** or in the presence of C3NeF IgGs purified from patients P5 **(C)** and P10 **(D)**, were further incubated with buffer alone (decay –) or with buffer added with 2,640 ng/ml FH (decay +), respectively, for 30, 60, 120, and 240 min at 37°C The percentage of residual B band was calculated as the ratio of the densities (in Pixel^2^) of each B band after decay and the corresponding baseline B band density before decay x 100 and results are reported in the bottom graphs. The membranes were then incubated with an anti-FH antibody and FH band could be visualized at 150 KDa (top). Results of a representative microplate/WB experiment of *n* = 3 are shown.

To evaluate whether C3NeF IgGs stabilized the alternative pathway C3 proconvertase C3bB, we modified the microplate/WB assay, exploiting the selective stabilization properties of Mn^2+^. C3bB was formed on C3b-coated wells in the presence of FB and Mn^2+^ buffer, and we detected by WB the B component of the C3bB(Mn^2+^) proconvertase recovered from wells. The kinetics of C3bB(Mn^2+^) assembly and decay and the effect of FH at physiological molar ratio with FB, are shown in Figures 9A,B. In these experimental conditions, no Bb band of the C3 convertase was observed either during assembly or after decay, since C3bBb(Mn^2+^) is highly unstable and once formed it rapidly dissociates (35).

C3bB(Mn^2+^) proconvertase did not spontaneously decay, as shown by the intensity of B band that did not change over 240 min incubation. FH band could be detected in the WB of the products detached from the wells after each time interval, indicating that FH binds C3bB. However, FH did not affect either C3bB(Mn^2+^) formation or decay (Figures 9A,B), suggesting that in our condition FH neither competed with FB for the interaction with C3b nor displaced FB from C3b.

We then repeated the above experiments using C3NeF IgGs from two patients (P5 and P10) selected for being strongly positive in stabilizing C3 convertase C3bBb(Mg^2+^). As shown in Figures 9C,D, C3NeF IgGs added during C3bB(Mn^2+^) assembly did not modify the intensity of the B band at any time point either in the absence or in the presence of FH.

Finally, we performed additional microplate/WB experiments by incubating C3b-coated wells with FB, FD in the presence of Ni^2+^ buffer, a condition that stabilizes both C3bB and C3bBb, as demonstrated by the detection of B and Bb bands on WB after 30 min assembly (Supplementary Figure 1D).

**Figure 10:**
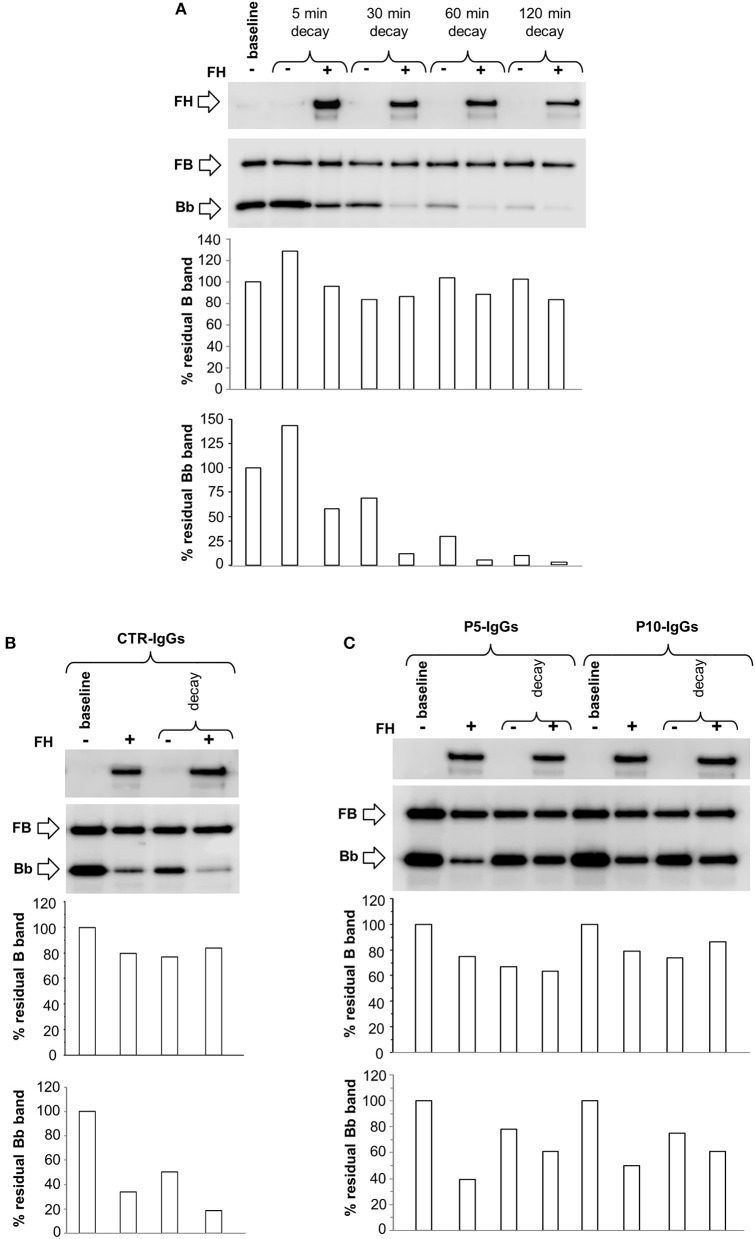
Formation and decay of C3bB(Ni^2+^) C3 proconvertase and C3bBb(Ni^2+^) C3 convertase in the absence or in the presence of patient IgGs, by microplate/WB assay. **(A)** The time course of spontaneous and FH-mediated decay of C3bB(Ni^2+^) and C3bBb(Ni^2+^), both formed during 30 min at 37°C (baseline) by incubation of C3b coated wells with FB (1,000 ng/ml), FD (10 ng/ml), and NiCl_2_ (2 mM), was monitored by further incubation at 37°C for 5, 30, 60, and 120 min with buffer alone (decay –) or buffer added with 2,640 ng/ml FH (decay +). **(B-C)** C3bB(Ni^2+^) and C3bBb(Ni^2+^) were formed for 30 min at 37°C in NiCl_2_ buffer without (baseline –) or with 2,640 ng/ml FH (+) in the presence of IgGs purified from an healthy subjects (CTR-IgGs, **B**) or from patients P5 and P10 (**C**, P5-IgGs and P10-IgGs). The complexes formed in the absence of FH were then allowed to decay for 30 min **(C)** at 37°C with buffer alone (decay –) or buffer added with 2,640 ng/ml FH (decay +). The percentage of residual B (93 KDa) or Bb (60 KDa) bands was calculated as the ratio of the densities (in Pixel^2^) of each B or Bb band after decay and the corresponding baseline B or Bb band density before decay x 100 and results are reported in the bottom graphs. The membranes were then incubated with an anti-FH antibody and FH band could be visualized at 150 KDa (top). Results of a representative microplate/WB experiment of *n* = 3 are shown.

The complexes were allowed to decay by incubation for different time points with buffer alone or buffer containing FH. We observed a slight spontaneous decay of C3bB(Ni^2+^) at 30 min, thereafter the intensity of B band remained stable (Figure 10A). FH did not affect C3bB(Ni^2+^) formation or decay (Figures 10A,B), confirming the results obtained in the Mn^2+^ ion selective conditions. At variance, FH reduced C3bBb(Ni^2+^) convertase formation and accelerated the decay, as shown by progressive disappearance of the Bb band on WB (Figure 10A).

Next, we assessed the effect of C3NeFs on the formation and decay of C3bB(Ni^2+^) and C3bBb(Ni^2+^). For this purpose, IgGs from P5 or P10 or from a healthy control were added to C3b-coated wells with FB and FD in the presence or in the absence of FH, followed by 30 min of decay. As observed in Mn^2+^ experiments, C3NeFs from P5 and P10 did not affect C3bB(Ni^2+^) assembly and decay substantially (Figures 10B,C). At variance, P5 and P10 IgGs strongly limited FH-mediated C3bBb(Ni^2+^) decay, and attenuated FH-mediated inhibitory effect on C3bBb(Mg^2+^) formation, as compared with control IgG (Figures 10B,C).

## Discussion

To characterize the diverse mechanisms through which the heterogeneous autoantibodies C3NeFs influence the function and regulation of the alternative pathway C3 convertase in primary IC-MPGN and C3G, we established a WB-based assay that monitors the formation and the spontaneous and regulated decay of the C3bBb convertase, through specific detection of the Bb band. The assay turned out to be very flexible and allowed us to study the effect of C3NeFs on spontaneous and/or FH-accelerated C3bBb decay, disclosing a subgroup of properdin-dependent C3NeFs and investigating the mechanisms underlying C3NeF activity. Finding that IgGs from all C3G/IC-MPGN controls without C3NeFs by hemolytic assay did not have C3 convertase stabilizing activity, supports the specificity of the new WB-based assays for analyzing C3NeFs.

Several methods have been reported in the literature for the evaluation of C3NeFs. Classic and modified hemolytic assays (26–28, 31, 40), performed on the surface of C3b-coated sheep erythrocytes are laborious and require expertise. In addition the read-out, based on the release of hemoglobin from lysed sheep erythrocytes, is an indirect measure of the terminal complement activation by residual C3 convertase sites on the cell surface and does not make it possible to directly evaluate the molecular mechanisms through which C3NeFs stabilize the C3bBb complex.

Functional molecular assays of C3NeFs have recently been reported. Surface Plasmon resonance with flowing C3NeFs, FB and FD over C3b immobilized on the chip allowed real-time analysis of C3 convertase stabilization (31), but this method is not universally available and required purified patients' IgG at high concentration.

Other, simpler ELISA-based assays have been described, in which C3b-coated microplate wells are incubated with FB plus FD in Mg^2+^ buffers, followed by detection with anti-FB antibodies. These assays detect the capacity of C3NeFs to stabilize the C3 convertase in the absence or in the presence of properdin (31). To analyze the effect C3NeFs on C3 convertase dissociation and on its accelerated decay mediated by FH and other complement regulators (31) the ELISA assays are done in the presence of Ni^2+^, which prolongs the half-life of C3bBb. However, this experimental condition does not distinguish between the alternative pathway C3 convertase and C3 proconvertase, because through WB analysis of the ELISA products of the reaction with Ni^2+^ in the presence of FD, here we visualized both the Bb and the B bands of C3bBb and C3bB complexes. The latter finding is consistent with earlier evidence that Ni^2+^ strongly stabilizes the proconvertase C3bB (41).

To overcome these issues, we took a step forward and modified the microplate assay, combining the selectivity of Mg^2+^ for C3bBb with the high sensitivity of the WB technique. In the absence of exogenous properdin, the Mg^2+^ microplate/WB assay revealed C3NeF IgGs capable of stabilizing C3bBb in 11 of 26 IC-MPGN or C3G patients. The finding that 9 of these C3NeFs had dual action, preventing both spontaneous and FH-accelerated C3 convertase decay, confirms published data that the large majority of C3NeFs also impair the decay-accelerating activity of regulatory proteins (25, 31). Since C3NeF IgGs were added during the first step of C3 convertase formation and unbound IgGs were washed out before the decay, the positivity in the microplate/WB test would suggest that C3 convertase-stabilizing IgGs rapidly and irreversibly bound to nascent C3bBb complexes. Essentially the same results were obtained when the assay was repeated adding C3NeF IgGs only during decay, indicating that C3 convertase-stabilizing IgGs also efficiently interacted with preformed C3bBb. One C3NeF IgG that was negative in the first protocol turned positive in the second setting, suggesting that it may act through reversible interaction with C3 convertase.

It is relevant that in the presence of exogenous properdin all but one patients' IgGs had C3 convertase-stabilizing activity, including IgGs negative in the assay without exogenous properdin, which confirms previous evidence that some C3NeFs require additional stability conferred by properdin to block C3 convertase decay (26, 28, 31). Properdin is the only known positive regulator of the alternative pathway of complement, and acts by extending the half-life of the C3 convertase 10-fold (42). It also facilitates the switch of the C3 convertase to the C5 convertase and stabilizes the C5 convertase complex (43, 44). The finding that C3NeFs with properdin-dependent C3 convertase-stabilizing activity associated with higher plasma sC5b-9 levels than C3NeFs with properdin-independent activity may be explained considering previous studies with hemolytic assays modified to interrogate C5 convertase stabilization, which demonstrated that in the presence of properdin a subset of C3NeFs enhances C5 cleavage and terminal pathway activation (28, 29, 45). In a cohort of patients with C3G Marinozzi et al. (28) found that plasma sC5b-9 levels correlated with C5 convertase-stabilizing activity of C3NeFs, which is consistent with our data. In addition, Paixao-Cavalcante et al. (31) showed that properdin-dependent C3NeFs enhanced C5 convertase activity while properdin-independent C3NeFs had no effect on C5 convertase activity. The former C3NeFs efficiently cleaved C3, but were “weak” C3 convertase binders and required the addition of properdin for the detection of their C3 convertase stabilizing activity (31).

We speculate that C3NeFs, which in the microplate/WB assay presented here were active in the assay without added properdin, recognize epitope(s) in the C3 convertase that are absent and/or masked in the C5 convertase complex, and cause C3-restricted complement activation *in vivo*, as reflected by low C3 and normal sC5b-9 levels. At variance properdin-dependent C3NeF would bind different epitopes that are present both in the C3 and the C5 convertase, resulting *in vivo* in complement activation until the terminal pathway with low C3 and high sC5b-9 levels.

Remarkably, we found that 6 of the 9 patients with DDD carried C3NeF IgGs that exerted C3 convertase-stabilizing activity also without properdin addition, and had normal sC5b-9 levels. In line with our data, using a modified hemolytic assay Zhang et al (26) reported that of 32 patients with DDD, 88% of those with C3NeF activity were positive in the assay without properdin. In another report (28), 63% of C3NeF-positive DDD patients had C3NeFs targeting the C3 convertase but not the C5 convertase.

As for properdin-dependent C3NeFs, we identified them mostly in patients with either C3GN [3 of 4] or primary IC-MPGN (9 of 13) and high plasma levels of sC5b-9. The association of properdin-dependent C3NeFs with C3GN and terminal complement pathway activation *in vivo* is consistent with recent findings that in most (67%) C3GN patients, C3NeFs target both the C3 and C5 convertases (28), and that circulating markers of terminal pathway activity are altered more in C3GN than in DDD (46). Another study documented low properdin levels in C3NeF negative C3G patients and serum properdin consumption was a marker of C5 convertase dysregulation, indeed properdin levels inversely correlated with sC5b-9 in plasma (47). Properdin levels were normal in C3G patients with C3NeF (47).

To the best of our knowledge, no other studies have formally explored the mechanisms of action of C3NeFs in IC-MPGN yet. Old reports proposed an association between properdin-dependent C3NeFs and MPGN type I and III (38, 39), but they antedated the current IF-based classification. Finding comparable properdin-dependency and comparable levels of C3 convertase-stabilizing activity in C3GN and primary IC-MPGN patients here would suggest the existence of a common pathogenetic mechanism that causes complement alternative pathway dysregulation in the two histology groups. We hypothesize that in IC-MPGN patients, disease was initiated by an as yet unidentified trigger (11, 12), causing glomerular immune-complex deposition and possibly the formation of C3NeF IgGs, the latter resulting in a switch from acute classical complement pathway-driven disease to chronic alternative pathway-driven disease. Although the IF-based classification of C3G/IC-MPGN is an advance toward an etiology-based approach of these diseases, it is likely too simplistic to rely only on C3 dominance for distinguishing patients with abnormalities of complement activation and those with immune complex-mediated disease, as suggested by a recent review (48). Our results highlight the need of a more advanced classification, based on the underlying pathogenetic patterns of complement activation. Along this line are data that the stratification of patients according to C3NeF activity was more clear-cut when we compared patients grouped on the basis of cluster analysis (15). Thus, patients in cluster 3, characterized by highly electron-dense intramembranous deposits, low C3 levels and mostly normal sC5b-9 levels, had a higher prevalence of C3NeFs stabilizing C3 convertase and higher stabilizing activity in the assay without properdin than patients in clusters 1 and 2. Instead, about 70% of patients in clusters 1 and 2, with a high prevalence of subendothelial, subepithelial and mesangial deposits, had properdin-dependent C3NeFs, which fits with low C3 levels and high sC5b-9 levels measured *in vivo* [(15) and present data]. Altogether these findings highlight two different mechanisms of complement dysregulation by C3NeFs, with different degrees of unchecked activity of the C3 and C5 convertases resulting in different complement profiles and different patterns of glomerular injury. Thus, prevalent dysregulation of the C3 convertase by properdin-independent C3NeFs may result in the constant, slow accumulation of C3 breakdown products in the GBM, generating denser intramembranous deposits. In patients with properdin-dependent C3NeFs, both C3 and C5 convertase dysregulation is high, and terminal complement activation products are sequestered in the glomerulus and participate in the formation of more amorphous deposits along the glomerular membrane layers. The significantly higher prevalence of nephrotic syndrome during disease course in patients with properdin-dependent C3NeFs supports the role of a pathogenetic mechanism involving the terminal complement pathway in altering glomerular permselectivity properties.

Another important observation from this study is that the stabilizing effect of both properdin-independent and properdin-dependent C3NeFs against FH-mediated C3 convertase decay is not due to competition between C3NeFs and FH, because in the microplate/WB assays the binding of FH to either nascent C3bBb, during the formation step, or to preformed C3bBb during the decay step, was not reduced in the presence of C3NeFs. This finding indicates that C3NeFs and FH bind different molecular domains in the C3bBb complexes, and may be relevant for further studies aimed at identifying the neopitope(s) recognized by the different C3NeFs, an issue that remains ill-defined (49). Considering that FH binds C3b (50), and is very efficient in Bb displacement from C3b (21), it is possible to speculate that C3NeFs could be directed against epitopes on Bb, as previously proposed (51).

Notably, the C3NeFs that stabilized C3 convertase against FH-accelerated decay also partially but significantly prevented the inhibitory effect of FH on C3 convertase formation. This effect could not be attributed to an antagonistic effect of C3NeFs with FH, since C3NeFs do not interact with the single C3 convertase component proteins, as demonstrated by earlier studies with C3b, FB, and Bb immobilized on microwell plates (31), nor do they prevent FH binding in the assays reported here. We hypothesize that C3NeFs rapidly stabilized C3 convertase to prevent the FH-induced dissociation of newly formed C3 convertase complexes.

Whether C3NeFs can have any effect on the complement alternative pathway C3 proconvertase enzyme is not known. To address this issue, two of the C3NeFs that exhibited the highest stabilizing activity on C3 convertase were tested in the microplate/WB assay with Mn^2+^ buffer, designed to selectively interrogate C3 proconvertase stabilization. C3bB complexes did not undergo FH-accelerated decay, which is consistent with other data in the literature (52, 53), and the addition of C3NeFs did not alter the amount of residual C3bB complexes. The results were not restricted to experiments with the Mn^2+^ buffer, because when we repeated the test in Ni^2+^ buffer, no effects of either FH or C3NeFs were observed on C3bB proconvertase decay, while in the same conditions FH dissociated the C3bBb C3 convertase and C3NeFs antagonized the FH-mediated decay-accelerating effect.

Intriguingly, we observed that independently of the ions (Mn^2+^ or Ni^2+^) used in the reaction buffer, C3 proconvertase complexes formed in the same amounts in the absence and in the presence of FH, suggesting that FH did not compete enough with FB for binding to C3b as to prevent C3bB assembly. A plausible explanation for these results derives from biophysical studies showing that the binding affinity between C3b and FH molecules is lower compared with the affinity between C3b and FB (35, 50). FH binding to negatively charged proteoglycans in cell glycocalyx and on cell surfaces shifts FH from a latent to an active conformation, which significantly increases the affinity for C3b (54). It is possible that in a cell-based context proteoglycans could influence the interactions of C3b with FB or FH in favor of the latter (55).

In summary, our study provides a mechanistic approach to detecting and characterizing C3NeFs in patients with C3G and IC-MPGN and identifies two distinct pathogenetic mechanisms through which C3NeFs may cause complement dysregulation and glomerular disease, confirming at molecular level previous studies with hemolytic and ELISA-based assays (28, 31). C3NeFs that stabilize C3 convertase in the assay without properdin associate with prevalent dysregulation of the C3 convertase and the formation of highly electron-dense deposits in the GBM, while properdin-dependent C3NeFs result in both C3 and C5 convertase dysregulation, high plasma sC5b-9 levels and more amorphous and broadly distributed glomerular deposits.

The identification of different types of C3NeFs with distinct functional specificities may have an impact on patient management. Our results suggest that a therapy inhibiting C5 activation (56–58) or targeting properdin (59) could potentially benefit patients with properdin-dependent C3NeFs. Patients with properdin-independent C3NeFs might benefit from emerging drugs, such as FD or FB inhibitors that target the C3 convertase of the complement alternative pathway (60).

Finally, results of mechanistic studies highlighted new information, which will be relevant to further studies aimed to clarify the way by which C3NeFs induce complement dysregulation: (1) most C3NeFs bind strongly and irreversibly to C3 convertase; (2) C3NeFs and FH recognize different epitopes in C3 convertase; (3) C3NeFs bind rapidly to C3 convertase and antagonize the decay-accelerating activity of FH on newly formed complexes; (4) C3NeFs do not affect the formation and stability of the C3 proconvertase.

## Author contributions

RD, MN, and GR designed research, interpreted data, and wrote the paper. RD, PP, RP, and EV performed the research and analyzed the data. PI collected clinical data and performed cluster analysis. AB analyzed the data and critically revised the manuscript.

### Conflict of interest statement

MN has received honoraria from Alexion Pharmaceuticals for giving lectures, and for participating in advisory boards and research grants from Omeros, Alnylam, and Chemocentryx. GR has consultancy agreements with AbbVie^*^, Alexion Pharmaceuticals^*^, Bayer Healthcare^*^, Reata Pharmaceuticals^*^, Novartis Pharma^*^, AstraZeneca^*^, Otsuka Pharmaceutical Europe^*^, Concert Pharmaceuticals^*^.

^*^No personal remuneration is accepted, compensation is paid to his institution for research and educational activities. None of these activities have had any influence on the results or interpretations in this article.

The remaining authors declare that the research was conducted in the absence of any commercial or financial relationships that could be construed as a potential conflict of interest.
