# Supplementary material for: Unraveling the Molecular Mechanisms Underlying Complement Dysregulation by Nephritic Factors in C3G and IC-MPGN
Source: Front Immunol. 2018 Oct 15;9:2329. doi: 10.3389/fimmu.2018.02329 (PMC6248175; doi:10.3389/fimmu.2018.02329)
Supplement: Supplementary file 1 [file Data_Sheet_1.pdf]

Supplementary material

Manuscript #383223 Revised version

UNRAVELLING THE MOLECULAR MECHANISMS UNDERLYING COMPLEMENT DYSREGULATION BY NEPHRITIC FACTORS IN C3G AND IC-MPGN

Roberta Donadelli, Patrizia Pulieri, Rossella Piras, Paraskevas Iatropoulos, Elisabetta Valoti, Ariela Benigni, Giuseppe Remuzzi, Marina Noris

Supplementary figures and tables

Supplementary figure 1

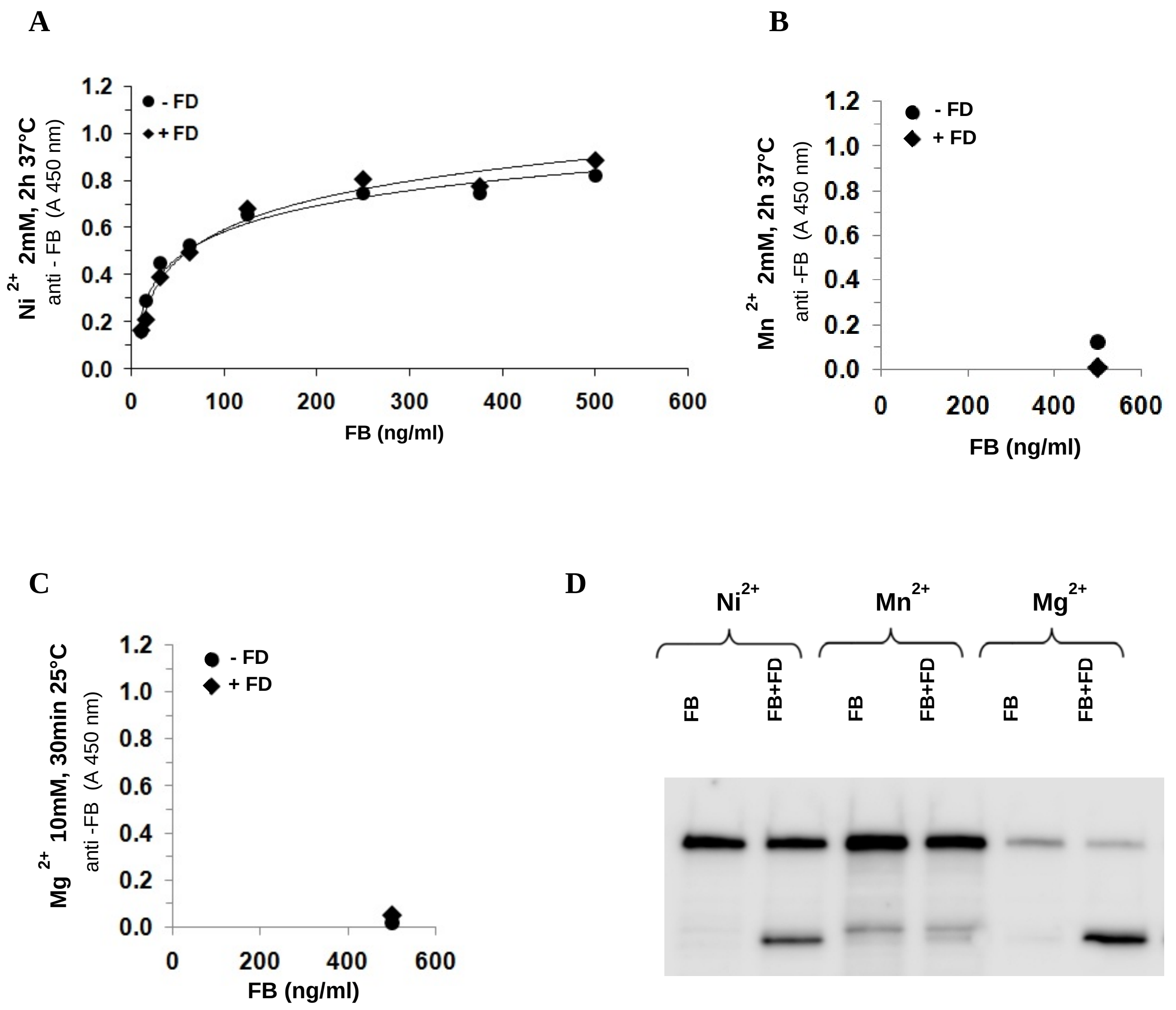

Supplementary Figure 1. **Detection of formation of C3bB C3 proconvertase and C3bBb C3 convertase complexes by ELISA and microplate/Western blot (WB) assays**. (**A**), Detection of Ni^{2+}-dependent C3bB and C3bBb. C3b-coated microtiter wells were incubated with increasing amounts of FB (0-500 ng/ml) and 2 mM $NiCl_2$ in the absence or in the presence of 25 ng/ml FD and analysed by ELISA by using an anti-FB antibody. (**B-C)** C3b coated wells were incubated with 500 ng/ml FB in the presence or in the absence of 25 ng/ml FD, 2 mM $MnCl_2$ or 10 mM $MgCl_2$ to obtain C3bB(Mn^{2+}) and C3bBb(Mg^{2+}), respectively. Complexes formed were detected by ELISA with an anti-FB antibody. (**D)** C3bB and C3bBb complexes were formed with $NiCl_2$, $MnCl_2$ or $MgCl_2$, then detached from the wells and analysed by WB with an anti-FB antibody. C3bB and C3bBb formation was evaluated by the visualization of the B band (93 KDa) and the Bb band (60 KDa), respectively. One representative experiment of three is shown.

Supplementary Figure 2.

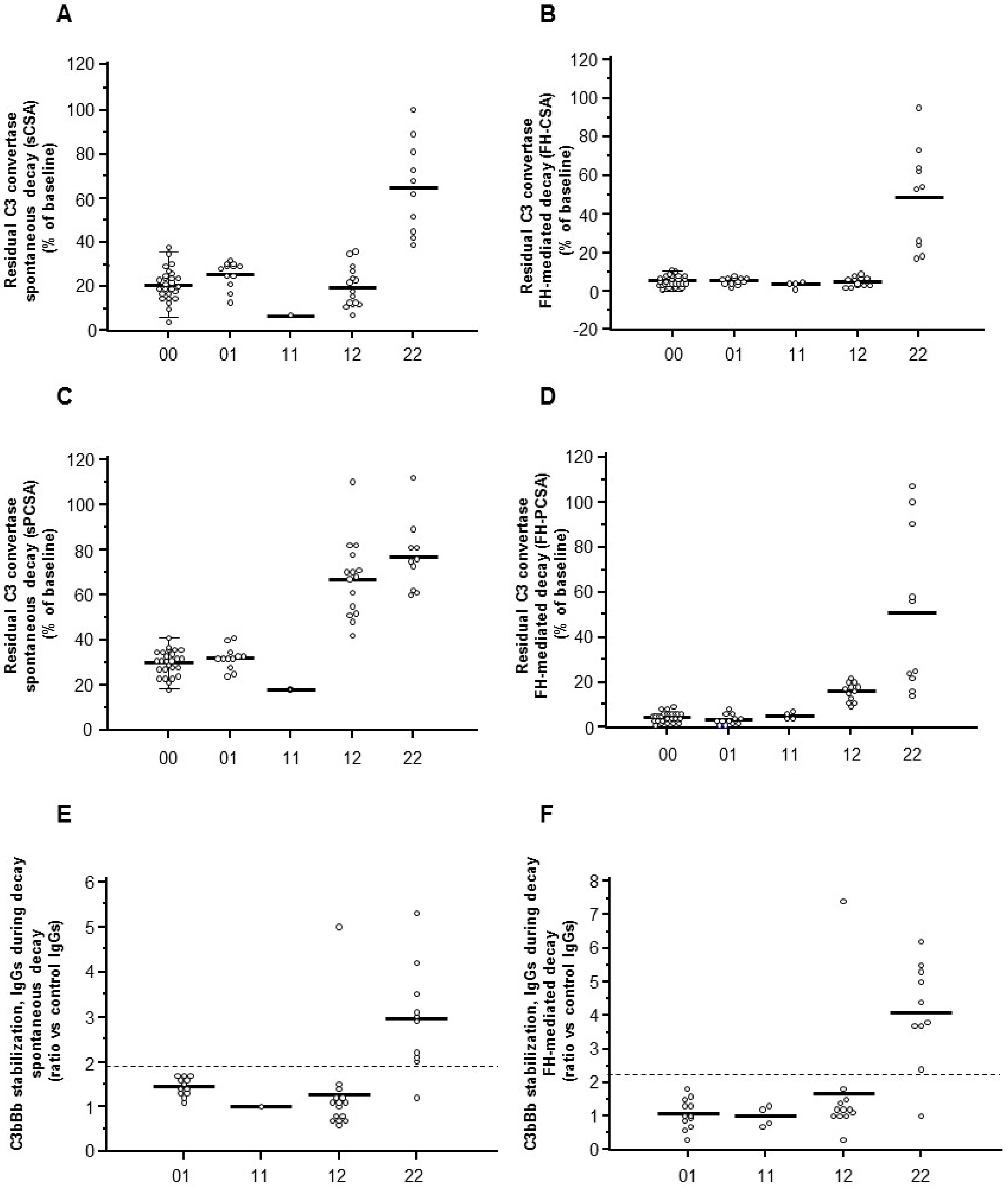

Supplementary Figure 2. **Individual values of residual C3 convertase after decay in healthy subjects, in control C3G/IC-MPGN patients negative for C3NeF by hemolytic assay (C3G/IC-MPGN controls) and in C3G/IC-MPGN patients with C3NeF by hemolytic assay (C3G/IC-MPGN cases)**

(**A-B) Protocol 1.** C3bBb(Mg^{2+}) complexes were formed by incubating at 25° C for 12 min C3b-coated wells with 1000 ng/ml FB, 10 ng/ml FD, 100 μg/ml IgGs purified from healthy donors, or C3G/IC-MPGN cases or C3G/IC-MPGN controls and 10 mM $MgCl_2$. Spontaneous (**A, sCSA**) or FH-mediated (**B, FH-CSA**) decay of the complexes was monitored by further incubation of C3bBb(Mg^{2+}) formed in the absence of FH for 32 min at 25° C with buffer alone or 2640 ng/ml FH respectively.

The percentage of residual Bb band (visualized by an anti-FB antibody) was calculated as the ratio of the densities (in $Pixel^2$) of each Bb band after decay and the corresponding baseline Bb band density before decay x 100.

(**C-D) Protocol 3.** C3bBb(Mg^{2+}) complexes were formed by incubating at 25° C for 12 min C3b-coated wells with 1000 ng/ml FB, 10 ng/ml FD, 100 μg/ml IgGs from from healthy donors, or C3G/IC-MPGN cases or C3G/IC-MPGN controls and 10 mM $MgCl_2$ in the presence of 500 ng/ml Properdin (P) (baseline). In additional wells after washing, spontaneous or FH-mediated decay of the formed complexes were monitored by further incubation for 32 min at 25° C with buffer alone (**sPCSA, C**) or buffer added with 2640 ng/ml FH (**FH-PCSA, D**), respectively.

(**E-F) Protocol 2.** C3bBb(Mg^{2+}) complexes were formed by incubating at 25° C for 12 min C3b-coated wells with 1000 ng/ml FB, 10 ng/ml FD, and 10 mM $MgCl_2$. The complexes formed were then incubated in the presence of 100 μg/ml IgGs from healthy donors, or C3G/IC-MPGN cases or C3G/IC-MPGN controls without (spontaneous decay**, E**) or with (FH-mediated decay**, F**) 2640 ng/ml FH. The C3NeF IgG C3 convertase-stabilizing activity was quantified as the ratio of the densities (in $pixel^2$) of Bb bands (visualized by an anti-FB antibody) after decay in the presence of patient IgGs/ Bb band after decay in the presence of control IgGs (ratio patient Bb band/control Bb band).

The percentage of residual Bb band was calculated as the ratio of the densities (in $Pixel^2$) of each Bb band (visualized by an anti-FB antibody) after decay and the corresponding baseline Bb band density before decay x 100.

00: healthy subjects, n=26
01: control C3G/IC-MPGN patients negative for C3NeF by hemolytic assay, n=12.
11: C3G/IC-MPGN cases without C3 convertase stabilizing activity (spontaneous decay n=1 panels A, C and E; FH-decay n=4, panels B, D and F).
12: C3G/IC-MPGN cases with properdin dependent C3 convertase stabilizing activity (spontaneous decay n=15 panels A,C and E; FH-decay n=12, panels B, D and F).
22: C3G/IC-MPGN cases with properdin independent C3 convertase stabilizing activity (spontaneous decay n=10 panels A, C and E; FH-decay n=10, panels B, D and F).
The horizontal bars through the plots are the means. Vertical lines in **00** groups in panels A-D indicate ±2SD. The dashed horizontal lines in panels E and F show the limit of positive values (ratios between the mean + 2SD and the mean of residual Bb band in the reactions with IgGs from 26 healthy subjects).

Supplementary Table 1. Clinical features, complement assessment, genetic screening and histologic features in patients classified according to the type of C3 convertase stabilizing activity (FH-mediated decay).

Variable	FH-CSA-/ FH-PCSA-	FH-CSA-/ FH-PCSA+	FH-CSA+/ FH-PCSA+	Overall p-value	- / + vs. + / + p-value
**N**	4	12	10		
**Gender (% males)**	0%	58%	70%	0.056	0.675
**Data at onset**					
**Age (y) - Mean (SD)**	13.1 (±8.2)	13.7 (±6.9)	11.1 (±3)	0.597	0.288
**Microhematuria**	75%	91%	90%	0.55	1.00
**Gross hematuria**	25%	17%	60%	0.127	0.074
**Proteinuria**	75%	100%	90%	0.142	0.455
**Nephrotic syndrome**	50%	42%	40%	1.00	1.00
**Renal impairment**	0%	8%	0%	1.00	1.00
**Familiarity for nephropathy**	0%	17%	0%	0.631	0.481
**Serum C3 (mg/dl)**	24.5 (±24.8)	25.4 (±14.8)	21.6 (±16.5)	0.870	0.575
**Serum C4 (mg/dl)**	15 (±9.2)	17.3 (±8.9)	24.4 (±8)	0.100	0.067
**Plasma sC5b-9 (ng/ml)**	1654 (±1276)	1718 (±1309)	616 (±612)	0.065	**0.024**
**Low serum C3 and normal serum C4**	50%	75%	100%	0.074	0.221
**C3NeF positive**	100%	100%	90%	0.540	0.46
**LPV carriers**	50%	17%	20%	0.393	1.00
Data during follow-up					
**Nephrotic syndrome**	75%	83%	40%	0.127	**0.074**
**High blood pressure**	0%	50%	67%	0.130	0.66
**Chronic kidney disease**	0%	33%	30%	0.704	1.00
**ESRD**	0%	8%	10%	1.00	1.00
**Histological features**					
**Time Onset to Biopsy (yr)**	1.6 (±2.3)	1.6 (±2.4)	0.7 (±1.2)	0.511	0.260
Light microscopy					
**Sclerotic glomeruli**	2% (±4%)	1% (±2%)	0% (±1%)	0.450	0.698
**Crescents**	6% (±11%)	1% (±2%)	15% (±30%)	0.256	0.125
**Degree of mesangial proliferation***	1.5 (±1.3)	1.2 (±1.2)	1.7 (±0.9)	0.585	0.302
**Degree of endocapillary proliferation***	0.2 (±0.5)	1.2 (±0.9)	1 (±1.1)	0.253	0.688
**Degree of interstitial inflammation***	1.2 (±1)	0.6 (±0.7)	0.4 (±0.5)	0.160	0.495
**Degree of interstitial fibrosis***	0.5 (±0.6)	0.4 (±0.7)	0.1 (±0.3)	0.440	0.320
**Degree of arteriolar sclerosis***	0 (±0)	0.1 (±0.3)	0.1 (±0.3)	0.815	0.888
Immunofluorescence					
**C3***	2.6 (±0.5)	2.8 (±0.3)	2.8 (±0.4)	0.614	0.923
**IgA***	1.0 (±0.8)	0.3 (±0.6)	0.1 (±0.2)	**0.025**	0.223
**IgG***	1.2 (±1.5)	1.8 (±1.3)	0.6 (±0.7)	0.090	**0.023**
**IgM***	1.6 (±0.8)	1.1 (±0.9)	0.8 (±0.8)	0.271	0.431
**C1q***	0.5 (±0.6)	1.2 (±1.1)	0.6 (±0.8)	0.198	0.130
**Fibrinogen***	0.9 (±1.4)	0.4 (±0.7)	0.1 (±0.3)	0.260	0.320
Electron microscopy					
**Mesangial deposits**	50%	55%	40%	0.866	0.670
**Subepithelial deposits**	50%	36%	30%	0.864	1.00
**Subepithelial hump-like deposits**	0%	22%	20%	1.00	1.00
**Subendothelial deposits**	75%	91%	30%	**0.010**	**0.008**
**Intramembranous granular deposits**	25%	73%	20%	**0.033**	**0.030**
**Intramembranous highly electron-dense deposits**	50%	9%	70%	**0.012**	**0.008**

*Degree of mesangial proliferation, endocapillary proliferation, interstitial inflammation, interstitial fibrosis, and arteriolar sclerosis, as well as IF findings were graded using a scale of 0 to 3, including 0, trace (0.5+), 1+, 2+ and 3+. Quantitative variables are expressed as mean (±S.D.) unless otherwise specified. Serum C3: reference 90-180 mg/dl; serum C4: reference 10-40 mg/dl; plasma

sC5b-9: reference ≤400 ng/ml. LPV: Likely pathogenetic variants. FH-CSA-/FH-PCSA-: patients without C3 convertase stabilizing activity; FH-CSA-/FH-PCSA+: patients with properdin-dependent C3 convertase stabilizing activity; FH-CSA+/FH-PCSA+: patients with properdin-independent C3 convertase stabilizing activity. Bold numbers indicate statistically significant values.
